# Strengthening the Reporting of Observational Studies in Epidemiology (STROBE): Explanation and Elaboration

**DOI:** 10.1371/journal.pmed.0040297

**Published:** 2007-10-16

**Authors:** Jan P Vandenbroucke, Erik von Elm, Douglas G Altman, Peter C Gøtzsche, Cynthia D Mulrow, Stuart J Pocock, Charles Poole, James J Schlesselman, Matthias Egger, for the STROBE Initiative

**Affiliations:** 1 Department of Clinical Epidemiology, Leiden University Medical Center, Leiden, The Netherlands; 2 Institute of Social & Preventive Medicine (ISPM), University of Bern, Bern, Switzerland; 3 Department of Medical Biometry and Medical Informatics, University Medical Centre, Freiburg, Germany; 4 Cancer Research UK/NHS Centre for Statistics in Medicine, Oxford, United Kingdom; 5 Nordic Cochrane Centre, Rigshospitalet, Copenhagen, Denmark; 6 University of Texas Health Science Center, San Antonio, United States of America; 7 Medical Statistics Unit, London School of Hygiene and Tropical Medicine, London, United Kingdom; 8 Department of Epidemiology, University of North Carolina School of Public Health, Chapel Hill, United States of America; 9 Department of Biostatistics, University of Pittsburgh Graduate School of Public Health, and University of Pittsburgh Cancer Institute, Pittsburgh, United States of America; 10 Department of Social Medicine, University of Bristol, Bristol, United Kingdom

## Abstract

Much medical research is observational. The reporting of observational studies is often of insufficient quality. Poor reporting hampers the assessment of the strengths and weaknesses of a study and the generalisability of its results. Taking into account empirical evidence and theoretical considerations, a group of methodologists, researchers, and editors developed the Strengthening the Reporting of Observational Studies in Epidemiology (STROBE) recommendations to improve the quality of reporting of observational studies. The STROBE Statement consists of a checklist of 22 items, which relate to the title, abstract, introduction, methods, results and discussion sections of articles. Eighteen items are common to cohort studies, case-control studies and cross-sectional studies and four are specific to each of the three study designs. The STROBE Statement provides guidance to authors about how to improve the reporting of observational studies and facilitates critical appraisal and interpretation of studies by reviewers, journal editors and readers. This explanatory and elaboration document is intended to enhance the use, understanding, and dissemination of the STROBE Statement. The meaning and rationale for each checklist item are presented. For each item, one or several published examples and, where possible, references to relevant empirical studies and methodological literature are provided. Examples of useful flow diagrams are also included. The STROBE Statement, this document, and the associated Web site (http://www.strobe-statement.org/) should be helpful resources to improve reporting of observational research.

## Abstract 2

In this explanatory and elaboration document Mattias Egger and colleagues provide the meaning and rationale of each checklist item on the STROBE Statement.

## Introduction

Rational health care practices require knowledge about the aetiology and pathogenesis, diagnosis, prognosis and treatment of diseases. Randomised trials provide valuable evidence about treatments and other interventions. However, much of clinical or public health knowledge comes from observational research [1]. About nine of ten research papers published in clinical speciality journals describe observational research [2,3].

### The STROBE Statement

Reporting of observational research is often not detailed and clear enough to assess the strengths and weaknesses of the investigation [4,5]. To improve the reporting of observational research, we developed a checklist of items that should be addressed: the Strengthening the Reporting of Observational Studies in Epidemiology (STROBE) Statement (Table 1). Items relate to title, abstract, introduction, methods, results and discussion sections of articles. The STROBE Statement has recently been published in several journals [6]. Our aim is to ensure clear presentation of what was planned, done, and found in an observational study. We stress that the recommendations are not prescriptions for setting up or conducting studies, nor do they dictate methodology or mandate a uniform presentation.

**Table 1:**
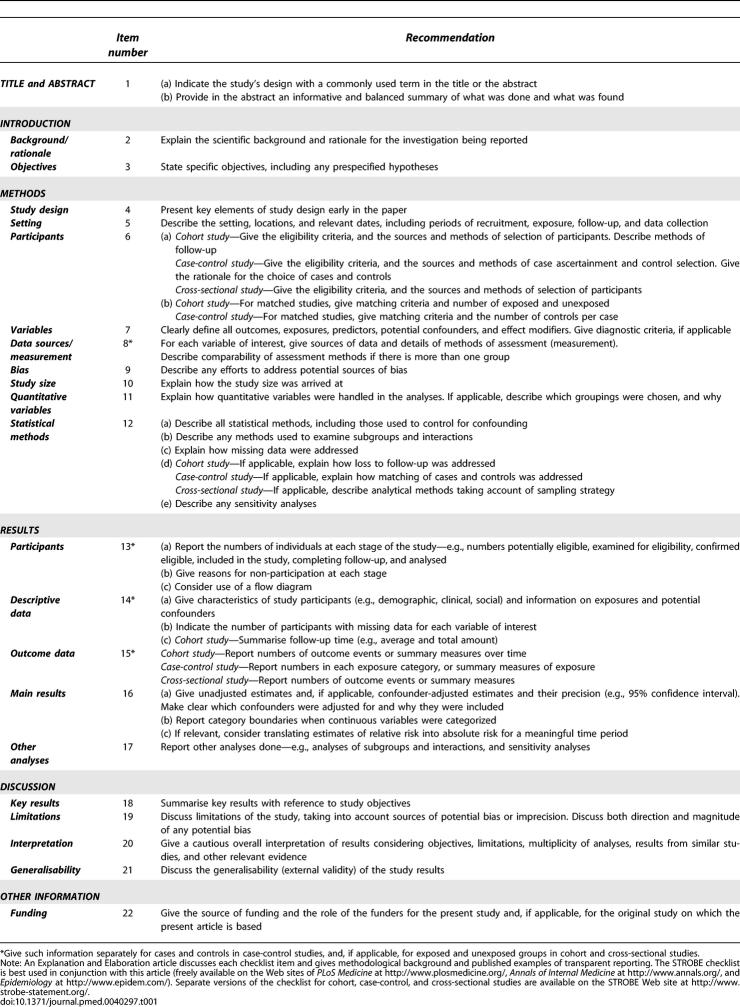
The STROBE Statement—Checklist of Items That Should Be Addressed in Reports of Observational Studies

STROBE provides general reporting recommendations for descriptive observational studies and studies that investigate associations between exposures and health outcomes. STROBE addresses the three main types of observational studies: cohort, case-control and cross-sectional studies. Authors use diverse terminology to describe these study designs. For instance, ‘follow-up study' and ‘longitudinal study' are used as synonyms for ‘cohort study', and ‘prevalence study' as synonymous with ‘cross-sectional study'. We chose the present terminology because it is in common use. Unfortunately, terminology is often used incorrectly [7] or imprecisely [8]. In Box 1 we describe the hallmarks of the three study designs.

### The Scope of Observational Research

Observational studies serve a wide range of purposes: from reporting a first hint of a potential cause of a disease, to verifying the magnitude of previously reported associations. Ideas for studies may arise from clinical observations or from biologic insight. Ideas may also arise from informal looks at data that lead to further explorations. Like a clinician who has seen thousands of patients, and notes one that strikes her attention, the researcher may note something special in the data. Adjusting for multiple looks at the data may not be possible or desirable [9], but further studies to confirm or refute initial observations are often needed [10]. Existing data may be used to examine new ideas about potential causal factors, and may be sufficient for rejection or confirmation. In other instances, studies follow that are specifically designed to overcome potential problems with previous reports. The latter studies will gather new data and will be planned for that purpose, in contrast to analyses of existing data. This leads to diverse viewpoints, e.g., on the merits of looking at subgroups or the importance of a predetermined sample size. STROBE tries to accommodate these diverse uses of observational research - from discovery to refutation or confirmation. Where necessary we will indicate in what circumstances specific recommendations apply.

### How to Use This Paper

This paper is linked to the shorter STROBE paper that introduced the items of the checklist in several journals [6], and forms an integral part of the STROBE Statement. Our intention is to explain how to report research well, not how research should be done. We offer a detailed explanation for each checklist item. Each explanation is preceded by an example of what we consider transparent reporting. This does not mean that the study from which the example was taken was uniformly well reported or well done; nor does it mean that its findings were reliable, in the sense that they were later confirmed by others: it only means that this particular item was well reported in that study. In addition to explanations and examples we included Boxes 1–8 with supplementary information. These are intended for readers who want to refresh their memories about some theoretical points, or be quickly informed about technical background details. A full understanding of these points may require studying the textbooks or methodological papers that are cited.

STROBE recommendations do not specifically address topics such as genetic linkage studies, infectious disease modelling or case reports and case series [11,12]. As many of the key elements in STROBE apply to these designs, authors who report such studies may nevertheless find our recommendations useful. For authors of observational studies that specifically address diagnostic tests, tumour markers and genetic associations, STARD [13], REMARK [14], and STREGA [15] recommendations may be particularly useful.

### The Items in the STROBE Checklist

We now discuss and explain the 22 items in the STROBE checklist (Table 1), and give published examples for each item. Some examples have been edited by removing citations or spelling out abbreviations. Eighteen items apply to all three study designs whereas four are design-specific. Starred items (for example item 8*) indicate that the information should be given separately for cases and controls in case-control studies, or exposed and unexposed groups in cohort and cross-sectional studies. We advise authors to address all items somewhere in their paper, but we do not prescribe a precise location or order. For instance, we discuss the reporting of results under a number of separate items, while recognizing that authors might address several items within a single section of text or in a table.

## The Items

### TITLE AND ABSTRACT 1 (a). Indicate the study's design with a commonly used term in the title or the abstract.

#### Example

“Leukaemia incidence among workers in the shoe and boot manufacturing industry: a case-control study” [18].

#### Explanation

Readers should be able to easily identify the design that was used from the title or abstract. An explicit, commonly used term for the study design also helps ensure correct indexing of articles in electronic databases [19,20].

### 1 (b). Provide in the abstract an informative and balanced summary of what was done and what was found.

#### Example

“**Background:** The expected survival of HIV-infected patients is of major public health interest.

**Objective:** To estimate survival time and age-specific mortality rates of an HIV-infected population compared with that of the general population.

**Design:** Population-based cohort study.

**Setting:** All HIV-infected persons receiving care in Denmark from 1995 to 2005.

**Patients:** Each member of the nationwide Danish HIV Cohort Study was matched with as many as 99 persons from the general population according to sex, date of birth, and municipality of residence.

**Measurements:** The authors computed Kaplan–Meier life tables with age as the time scale to estimate survival from age 25 years. Patients with HIV infection and corresponding persons from the general population were observed from the date of the patient's HIV diagnosis until death, emigration, or 1 May 2005.

**Results:** 3990 HIV-infected patients and 379 872 persons from the general population were included in the study, yielding 22 744 (median, 5.8 y/person) and 2 689 287 (median, 8.4 years/person) person-years of observation. Three percent of participants were lost to follow-up. From age 25 years, the median survival was 19.9 years (95% CI, 18.5 to 21.3) among patients with HIV infection and 51.1 years (CI, 50.9 to 51.5) among the general population. For HIV-infected patients, survival increased to 32.5 years (CI, 29.4 to 34.7) during the 2000 to 2005 period. In the subgroup that excluded persons with known hepatitis C coinfection (16%), median survival was 38.9 years (CI, 35.4 to 40.1) during this same period. The relative mortality rates for patients with HIV infection compared with those for the general population decreased with increasing age, whereas the excess mortality rate increased with increasing age.

**Limitations:** The observed mortality rates are assumed to apply beyond the current maximum observation time of 10 years.

**Conclusions:** The estimated median survival is more than 35 years for a young person diagnosed with HIV infection in the late highly active antiretroviral therapy era. However, an ongoing effort is still needed to further reduce mortality rates for these persons compared with the general population” [21].

#### Explanation

The abstract provides key information that enables readers to understand a study and decide whether to read the article. Typical components include a statement of the research question, a short description of methods and results, and a conclusion [22]. Abstracts should summarize key details of studies and should only present information that is provided in the article. We advise presenting key results in a numerical form that includes numbers of participants, estimates of associations and appropriate measures of variability and uncertainty (e.g., odds ratios with confidence intervals). We regard it insufficient to state only that an exposure is or is not significantly associated with an outcome.

A series of headings pertaining to the background, design, conduct, and analysis of a study may help readers acquire the essential information rapidly [23]. Many journals require such structured abstracts, which tend to be of higher quality and more readily informative than unstructured summaries [24,25].

**Box 1. Main study designs covered by STROBE**

Cohort, case-control, and cross-sectional designs represent different approaches of investigating the occurrence of health-related events in a given population and time period. These studies may address many types of health-related events, including disease or disease remission, disability or complications, death or survival, and the occurrence of risk factors.

In **cohort studies**, the investigators follow people over time. They obtain information about people and their exposures at baseline, let time pass, and then assess the occurrence of outcomes. Investigators commonly make contrasts between individuals who are exposed and not exposed or among groups of individuals with different categories of exposure. Investigators may assess several different outcomes, and examine exposure and outcome variables at multiple points during follow-up. Closed cohorts (for example birth cohorts) enrol a defined number of participants at study onset and follow them from that time forward, often at set intervals up to a fixed end date. In open cohorts the study population is dynamic: people enter and leave the population at different points in time (for example inhabitants of a town). Open cohorts change due to deaths, births, and migration, but the composition of the population with regard to variables such as age and gender may remain approximately constant, especially over a short period of time. In a closed cohort cumulative incidences (risks) and incidence rates can be estimated; when exposed and unexposed groups are compared, this leads to risk ratio or rate ratio estimates. Open cohorts estimate incidence rates and rate ratios.

In **case-control studies**, investigators compare exposures between people with a particular disease outcome (cases) and people without that outcome (controls). Investigators aim to collect cases and controls that are representative of an underlying cohort or a cross-section of a population. That population can be defined geographically, but also more loosely as the catchment area of health care facilities. The case sample may be 100% or a large fraction of available cases, while the control sample usually is only a small fraction of the people who do not have the pertinent outcome. Controls represent the cohort or population of people from which the cases arose. Investigators calculate the ratio of the odds of exposures to putative causes of the disease among cases and controls (see Box 7). Depending on the sampling strategy for cases and controls and the nature of the population studied, the odds ratio obtained in a case-control study is interpreted as the risk ratio, rate ratio or (prevalence) odds ratio [16,17]. The majority of published case-control studies sample open cohorts and so allow direct estimations of rate ratios.

In **cross-sectional studies**, investigators assess all individuals in a sample at the same point in time, often to examine the prevalence of exposures, risk factors or disease. Some cross-sectional studies are analytical and aim to quantify potential causal associations between exposures and disease. Such studies may be analysed like a cohort study by comparing disease prevalence between exposure groups. They may also be analysed like a case-control study by comparing the odds of exposure between groups with and without disease. A difficulty that can occur in any design but is particularly clear in cross-sectional studies is to establish that an exposure preceded the disease, although the time order of exposure and outcome may sometimes be clear. In a study in which the exposure variable is congenital or genetic, for example, we can be confident that the exposure preceded the disease, even if we are measuring both at the same time.

### INTRODUCTION

The Introduction section should describe why the study was done and what questions and hypotheses it addresses. It should allow others to understand the study's context and judge its potential contribution to current knowledge.

### 2. Background/rationale: Explain the scientific background and rationale for the investigation being reported.

#### Example

“Concerns about the rising prevalence of obesity in children and adolescents have focused on the well documented associations between childhood obesity and increased cardiovascular risk and mortality in adulthood. Childhood obesity has considerable social and psychological consequences within childhood and adolescence, yet little is known about social, socioeconomic, and psychological consequences in adult life. A recent systematic review found no longitudinal studies on the outcomes of childhood obesity other than physical health outcomes and only two longitudinal studies of the socioeconomic effects of obesity in adolescence. Gortmaker et al. found that US women who had been obese in late adolescence in 1981 were less likely to be married and had lower incomes seven years later than women who had not been overweight, while men who had been overweight were less likely to be married. Sargent et al. found that UK women, but not men, who had been obese at 16 years in 1974 earned 7.4% less than their non-obese peers at age 23. (…) We used longitudinal data from the 1970 British birth cohort to examine the adult socioeconomic, educational, social, and psychological outcomes of childhood obesity” [26].

#### Explanation

The scientific background of the study provides important context for readers. It sets the stage for the study and describes its focus. It gives an overview of what is known on a topic and what gaps in current knowledge are addressed by the study. Background material should note recent pertinent studies and any systematic reviews of pertinent studies.

### 3. Objectives: State specific objectives, including any prespecified hypotheses.

#### Example

“Our primary objectives were to 1) determine the prevalence of domestic violence among female patients presenting to four community-based, primary care, adult medicine practices that serve patients of diverse socioeconomic background and 2) identify demographic and clinical differences between currently abused patients and patients not currently being abused ” [27].

#### Explanation

Objectives are the detailed aims of the study. Well crafted objectives specify populations, exposures and outcomes, and parameters that will be estimated. They may be formulated as specific hypotheses or as questions that the study was designed to address. In some situations objectives may be less specific, for example, in early discovery phases. Regardless, the report should clearly reflect the investigators' intentions. For example, if important subgroups or additional analyses were not the original aim of the study but arose during data analysis, they should be described accordingly (see also items 4, 17 and 20).

### METHODS

The Methods section should describe what was planned and what was done in sufficient detail to allow others to understand the essential aspects of the study, to judge whether the methods were adequate to provide reliable and valid answers, and to assess whether any deviations from the original plan were reasonable.

### 4. Study design: Present key elements of study design early in the paper.

#### Example

“We used a case-crossover design, a variation of a case-control design that is appropriate when a brief exposure (driver's phone use) causes a transient rise in the risk of a rare outcome (a crash). We compared a driver's use of a mobile phone at the estimated time of a crash with the same driver's use during another suitable time period. Because drivers are their own controls, the design controls for characteristics of the driver that may affect the risk of a crash but do not change over a short period of time. As it is important that risks during control periods and crash trips are similar, we compared phone activity during the hazard interval (time immediately before the crash) with phone activity during control intervals (equivalent times during which participants were driving but did not crash) in the previous week” [28].

#### Explanation

We advise presenting key elements of study design early in the methods section (or at the end of the introduction) so that readers can understand the basics of the study. For example, authors should indicate that the study was a cohort study, which followed people over a particular time period, and describe the group of persons that comprised the cohort and their exposure status. Similarly, if the investigation used a case-control design, the cases and controls and their source population should be described. If the study was a cross-sectional survey, the population and the point in time at which the cross-section was taken should be mentioned. When a study is a variant of the three main study types, there is an additional need for clarity. For instance, for a case-crossover study, one of the variants of the case-control design, a succinct description of the principles was given in the example above [28].

We recommend that authors refrain from simply calling a study ‘prospective' or ‘retrospective' because these terms are ill defined [29]. One usage sees cohort and prospective as synonymous and reserves the word retrospective for case-control studies [30]. A second usage distinguishes prospective and retrospective cohort studies according to the timing of data collection relative to when the idea for the study was developed [31]. A third usage distinguishes prospective and retrospective case-control studies depending on whether the data about the exposure of interest existed when cases were selected [32]. Some advise against using these terms [33], or adopting the alternatives ‘concurrent' and ‘historical' for describing cohort studies [34]. In STROBE, we do not use the words prospective and retrospective, nor alternatives such as concurrent and historical. We recommend that, whenever authors use these words, they define what they mean. Most importantly, we recommend that authors describe exactly how and when data collection took place.

The first part of the methods section might also be the place to mention whether the report is one of several from a study. If a new report is in line with the original aims of the study, this is usually indicated by referring to an earlier publication and by briefly restating the salient features of the study. However, the aims of a study may also evolve over time. Researchers often use data for purposes for which they were not originally intended, including, for example, official vital statistics that were collected primarily for administrative purposes, items in questionnaires that originally were only included for completeness, or blood samples that were collected for another purpose. For example, the Physicians' Health Study, a randomized controlled trial of aspirin and carotene, was later used to demonstrate that a point mutation in the factor V gene was associated with an increased risk of venous thrombosis, but not of myocardial infarction or stroke [35]. The secondary use of existing data is a creative part of observational research and does not necessarily make results less credible or less important. However, briefly restating the original aims might help readers understand the context of the research and possible limitations in the data.

### 5. Setting: Describe the setting, locations, and relevant dates, including periods of recruitment, exposure, follow-up, and data collection.

#### Example

“The Pasitos Cohort Study recruited pregnant women from Women, Infant and Child clinics in Socorro and San Elizario, El Paso County, Texas and maternal-child clinics of the Mexican Social Security Institute in Ciudad Juarez, Mexico from April 1998 to October 2000. At baseline, prior to the birth of the enrolled cohort children, staff interviewed mothers regarding the household environment. In this ongoing cohort study, we target follow-up exams at 6-month intervals beginning at age 6 months” [36].

#### Explanation

Readers need information on setting and locations to assess the context and generalisability of a study's results. Exposures such as environmental factors and therapies can change over time. Also, study methods may evolve over time. Knowing when a study took place and over what period participants were recruited and followed up places the study in historical context and is important for the interpretation of results.

Information about setting includes recruitment sites or sources (e.g., electoral roll, outpatient clinic, cancer registry, or tertiary care centre). Information about location may refer to the countries, towns, hospitals or practices where the investigation took place. We advise stating dates rather than only describing the length of time periods. There may be different sets of dates for exposure, disease occurrence, recruitment, beginning and end of follow-up, and data collection. Of note, nearly 80% of 132 reports in oncology journals that used survival analysis included the starting and ending dates for accrual of patients, but only 24% also reported the date on which follow-up ended [37].

### 6. Participants: 6 (a). Cohort study: Give the eligibility criteria, and the sources and methods of selection of participants. Describe methods of follow-up.

#### Example

“Participants in the Iowa Women's Health Study were a random sample of all women ages 55 to 69 years derived from the state of Iowa automobile driver's license list in 1985, which represented approximately 94% of Iowa women in that age group. (…) Follow-up questionnaires were mailed in October 1987 and August 1989 to assess vital status and address changes. (…) Incident cancers, except for nonmelanoma skin cancers, were ascertained by the State Health Registry of Iowa (…). The Iowa Women's Health Study cohort was matched to the registry with combinations of first, last, and maiden names, zip code, birthdate, and social security number” [38].

### 6 (a). Case-control study: Give the eligibility criteria, and the sources and methods of case ascertainment and control selection. Give the rationale for the choice of cases and controls.

#### Example

“Cutaneous melanoma cases diagnosed in 1999 and 2000 were ascertained through the Iowa Cancer Registry (…). Controls, also identified through the Iowa Cancer Registry, were colorectal cancer patients diagnosed during the same time. Colorectal cancer controls were selected because they are common and have a relatively long survival, and because arsenic exposure has not been conclusively linked to the incidence of colorectal cancer” [39].

### 6 (a). Cross-sectional study: Give the eligibility criteria, and the sources and methods of selection of participants.

#### Example

“We retrospectively identified patients with a principal diagnosis of myocardial infarction (code 410) according to the International Classification of Diseases, 9th Revision, Clinical Modification, from codes designating discharge diagnoses, excluding the codes with a fifth digit of 2, which designates a subsequent episode of care (…) A random sample of the entire Medicare cohort with myocardial infarction from February 1994 to July 1995 was selected (…) To be eligible, patients had to present to the hospital after at least 30 minutes but less than 12 hours of chest pain and had to have ST-segment elevation of at least 1 mm on two contiguous leads on the initial electrocardiogram” [40].

#### Explanation

Detailed descriptions of the study participants help readers understand the applicability of the results. Investigators usually restrict a study population by defining clinical, demographic and other characteristics of eligible participants. Typical eligibility criteria relate to age, gender, diagnosis and comorbid conditions. Despite their importance, eligibility criteria often are not reported adequately. In a survey of observational stroke research, 17 of 49 reports (35%) did not specify eligibility criteria [5].

Eligibility criteria may be presented as inclusion and exclusion criteria, although this distinction is not always necessary or useful. Regardless, we advise authors to report all eligibility criteria and also to describe the group from which the study population was selected (e.g., the general population of a region or country), and the method of recruitment (e.g., referral or self-selection through advertisements).

Knowing details about follow-up procedures, including whether procedures minimized non-response and loss to follow-up and whether the procedures were similar for all participants, informs judgments about the validity of results. For example, in a study that used IgM antibodies to detect acute infections, readers needed to know the interval between blood tests for IgM antibodies so that they could judge whether some infections likely were missed because the interval between blood tests was too long [41]. In other studies where follow-up procedures differed between exposed and unexposed groups, readers might recognize substantial bias due to unequal ascertainment of events or differences in non-response or loss to follow-up [42]. Accordingly, we advise that researchers describe the methods used for following participants and whether those methods were the same for all participants, and that they describe the completeness of ascertainment of variables (see also item 14).

In case-control studies, the choice of cases and controls is crucial to interpreting the results, and the method of their selection has major implications for study validity. In general, controls should reflect the population from which the cases arose. Various methods are used to sample controls, all with advantages and disadvantages: for cases that arise from a general population, population roster sampling, random digit dialling, neighbourhood or friend controls are used. Neighbourhood or friend controls may present intrinsic matching on exposure [17]. Controls with other diseases may have advantages over population-based controls, in particular for hospital-based cases, because they better reflect the catchment population of a hospital, have greater comparability of recall and ease of recruitment. However, they can present problems if the exposure of interest affects the risk of developing or being hospitalized for the control condition(s) [43,44]. To remedy this problem often a mixture of the best defensible control diseases is used [45].

### 6 (b). Cohort study: For matched studies, give matching criteria and number of exposed and unexposed.

#### Example

“For each patient who initially received a statin, we used propensity-based matching to identify one control who did not receive a statin according to the following protocol. First, propensity scores were calculated for each patient in the entire cohort on the basis of an extensive list of factors potentially related to the use of statins or the risk of sepsis. Second, each statin user was matched to a smaller pool of non-statin-users by sex, age (plus or minus 1 year), and index date (plus or minus 3 months). Third, we selected the control with the closest propensity score (within 0.2 SD) to each statin user in a 1:1 fashion and discarded the remaining controls.” [46].

### 6 (b). Case-control study: For matched studies, give matching criteria and the number of controls per case.

#### Example

“We aimed to select five controls for every case from among individuals in the study population who had no diagnosis of autism or other pervasive developmental disorders (PDD) recorded in their general practice record and who were alive and registered with a participating practice on the date of the PDD diagnosis in the case. Controls were individually matched to cases by year of birth (up to 1 year older or younger), sex, and general practice. For each of 300 cases, five controls could be identified who met all the matching criteria. For the remaining 994, one or more controls was excluded...” [47].

#### Explanation

Matching is much more common in case-control studies, but occasionally, investigators use matching in cohort studies to make groups comparable at the start of follow-up. Matching in cohort studies makes groups directly comparable for potential confounders and presents fewer intricacies than with case-control studies. For example, it is not necessary to take the matching into account for the estimation of the relative risk [48]. Because matching in cohort studies may increase statistical precision investigators might allow for the matching in their analyses and thus obtain narrower confidence intervals.

In case-control studies matching is done to increase a study's efficiency by ensuring similarity in the distribution of variables between cases and controls, in particular the distribution of potential confounding variables [48,49]. Because matching can be done in various ways, with one or more controls per case, the rationale for the choice of matching variables and the details of the method used should be described. Commonly used forms of matching are frequency matching (also called group matching) and individual matching. In frequency matching, investigators choose controls so that the distribution of matching variables becomes identical or similar to that of cases. Individual matching involves matching one or several controls to each case. Although intuitively appealing and sometimes useful, matching in case-control studies has a number of disadvantages, is not always appropriate, and needs to be taken into account in the analysis (see Box 2).

Even apparently simple matching procedures may be poorly reported. For example, authors may state that controls were matched to cases ‘within five years', or using ‘five year age bands'. Does this mean that, if a case was 54 years old, the respective control needed to be in the five-year age band 50 to 54, or aged 49 to 59, which is within five years of age 54? If a wide (e.g., 10-year) age band is chosen, there is a danger of residual confounding by age (see also Box 4), for example because controls may then be younger than cases on average.

### 7. Variables: Clearly define all outcomes, exposures, predictors, potential confounders, and effect modifiers. Give diagnostic criteria, if applicable.

#### Example

“Only major congenital malformations were included in the analyses. Minor anomalies were excluded according to the exclusion list of European Registration of Congenital Anomalies (EUROCAT). If a child had more than one major congenital malformation of one organ system, those malformations were treated as one outcome in the analyses by organ system (…) In the statistical analyses, factors considered potential confounders were maternal age at delivery and number of previous parities. Factors considered potential effect modifiers were maternal age at reimbursement for antiepileptic medication and maternal age at delivery” [55].

#### Explanation

Authors should define all variables considered for and included in the analysis, including outcomes, exposures, predictors, potential confounders and potential effect modifiers. Disease outcomes require adequately detailed description of the diagnostic criteria. This applies to criteria for cases in a case-control study, disease events during follow-up in a cohort study and prevalent disease in a cross-sectional study. Clear definitions and steps taken to adhere to them are particularly important for any disease condition of primary interest in the study.

For some studies, ‘determinant' or ‘predictor' may be appropriate terms for exposure variables and outcomes may be called ‘endpoints'. In multivariable models, authors sometimes use ‘dependent variable' for an outcome and ‘independent variable' or ‘explanatory variable' for exposure and confounding variables. The latter is not precise as it does not distinguish exposures from confounders.

If many variables have been measured and included in exploratory analyses in an early discovery phase, consider providing a list with details on each variable in an appendix, additional table or separate publication. Of note, the *International Journal of Epidemiology* recently launched a new section with ‘cohort profiles', that includes detailed information on what was measured at different points in time in particular studies [56,57]. Finally, we advise that authors declare all ‘candidate variables' considered for statistical analysis, rather than selectively reporting only those included in the final models (see also item 16a) [58,59].

**Box 2. Matching in case-control studies**

In any case-control study, sensible choices need to be made on whether to use matching of controls to cases, and if so, what variables to match on, the precise method of matching to use, and the appropriate method of statistical analysis. Not to match at all may mean that the distribution of some key potential confounders (e.g., age, sex) is radically different between cases and controls. Although this could be adjusted for in the analysis there could be a major loss in statistical efficiency.

The use of matching in case-control studies and its interpretation are fraught with difficulties, especially if matching is attempted on several risk factors, some of which may be linked to the exposure of prime interest [50,51]. For example, in a case-control study of myocardial infarction and oral contraceptives nested in a large pharmaco-epidemiologic data base, with information about thousands of women who are available as potential controls, investigators may be tempted to choose matched controls who had similar levels of risk factors to each case of myocardial infarction. One objective is to adjust for factors that might influence the prescription of oral contraceptives and thus to control for *confounding by indication*. However, the result will be a control group that is *no longer representative* of the oral contraceptive use in the source population: controls will be older than the source population because patients with myocardial infarction tend to be older. This has several implications. A crude analysis of the data will produce odds ratios that are usually biased towards unity if the matching factor is associated with the exposure. The solution is to perform a matched or stratified analysis (see item 12d). In addition, because the matched control group ceases to be representative for the population at large, the exposure distribution among the controls can no longer be used to estimate the population attributable fraction (see Box 7) [52]. Also, the effect of the matching factor can no longer be studied, and the search for well-matched controls can be cumbersome – making a design with a non-matched control group preferable because the non-matched controls will be easier to obtain and the control group can be larger. *Overmatching* is another problem, which may reduce the efficiency of matched case-control studies, and, in some situations, introduce bias. Information is lost and the power of the study is reduced if the matching variable is closely associated with the exposure. Then many individuals in the same matched sets will tend to have identical or similar levels of exposures and therefore not contribute relevant information. Matching will introduce irremediable bias if the matching variable is not a confounder but in the causal pathway between exposure and disease. For example, in vitro fertilization is associated with an increased risk of perinatal death, due to an increase in multiple births and low birth weight infants [53]. Matching on plurality or birth weight will bias results towards the null, and this cannot be remedied in the analysis.

Matching is intuitively appealing, but the complexities involved have led methodologists to advise against routine matching in case-control studies. They recommend instead a careful and judicious consideration of each potential matching factor, recognizing that it could instead be measured and used as an adjustment variable without matching on it. In response, there has been a reduction in the number of matching factors employed, an increasing use of frequency matching, which avoids some of the problems discussed above, and more case-control studies with no matching at all [54]. Matching remains most desirable, or even necessary, when the distributions of the confounder (e.g., age) might differ radically between the unmatched comparison groups [48,49].

### 8. Data sources/measurement: For each variable of interest give sources of data and details of methods of assessment (measurement). Describe comparability of assessment methods if there is more than one group.

#### Example 1

“Total caffeine intake was calculated primarily using US Department of Agriculture food composition sources. In these calculations, it was assumed that the content of caffeine was 137 mg per cup of coffee, 47 mg per cup of tea, 46 mg per can or bottle of cola beverage, and 7 mg per serving of chocolate candy. This method of measuring (caffeine) intake was shown to be valid in both the NHS I cohort and a similar cohort study of male health professionals (...) Self-reported diagnosis of hypertension was found to be reliable in the NHS I cohort” [60].

#### Example 2

“Samples pertaining to matched cases and controls were always analyzed together in the same batch and laboratory personnel were unable to distinguish among cases and controls” [61].

#### Explanation

The way in which exposures, confounders and outcomes were measured affects the reliability and validity of a study. Measurement error and misclassification of exposures or outcomes can make it more difficult to detect cause-effect relationships, or may produce spurious relationships. Error in measurement of potential confounders can increase the risk of residual confounding [62,63]. It is helpful, therefore, if authors report the findings of any studies of the validity or reliability of assessments or measurements, including details of the reference standard that was used. Rather than simply citing validation studies (as in the first example), we advise that authors give the estimated validity or reliability, which can then be used for measurement error adjustment or sensitivity analyses (see items 12e and 17).

In addition, it is important to know if groups being compared differed with respect to the way in which the data were collected. This may be important for laboratory examinations (as in the second example) and other situations. For instance, if an interviewer first questions all the cases and then the controls, or vice versa, bias is possible because of the learning curve; solutions such as randomising the order of interviewing may avoid this problem. Information bias may also arise if the compared groups are not given the same diagnostic tests or if one group receives more tests of the same kind than another (see also item 9).

### 9. Bias: Describe any efforts to address potential sources of bias.

#### Example 1

“In most case-control studies of suicide, the control group comprises living individuals but we decided to have a control group of people who had died of other causes (…). With a control group of deceased individuals, the sources of information used to assess risk factors are informants who have recently experienced the death of a family member or close associate - and are therefore more comparable to the sources of information in the suicide group than if living controls were used” [64].

#### Example 2

“Detection bias could influence the association between Type 2 diabetes mellitus (T2DM) and primary open-angle glaucoma (POAG) if women with T2DM were under closer ophthalmic surveillance than women without this condition. We compared the mean number of eye examinations reported by women with and without diabetes. We also recalculated the relative risk for POAG with additional control for covariates associated with more careful ocular surveillance (a self-report of cataract, macular degeneration, number of eye examinations, and number of physical examinations)” [65].

#### Explanation

Biased studies produce results that differ systematically from the truth (see also Box 3). It is important for a reader to know what measures were taken during the conduct of a study to reduce the potential of bias. Ideally, investigators carefully consider potential sources of bias when they plan their study. At the stage of reporting, we recommend that authors always assess the likelihood of relevant biases. Specifically, the direction and magnitude of bias should be discussed and, if possible, estimated. For instance, in case-control studies information bias can occur, but may be reduced by selecting an appropriate control group, as in the first example [64]. Differences in the medical surveillance of participants were a problem in the second example [65]. Consequently, the authors provide more detail about the additional data they collected to tackle this problem. When investigators have set up quality control programs for data collection to counter a possible “drift” in measurements of variables in longitudinal studies, or to keep variability at a minimum when multiple observers are used, these should be described.

Unfortunately, authors often do not address important biases when reporting their results. Among 43 case-control and cohort studies published from 1990 to 1994 that investigated the risk of second cancers in patients with a history of cancer, medical surveillance bias was mentioned in only 5 articles [66]. A survey of reports of mental health research published during 1998 in three psychiatric journals found that only 13% of 392 articles mentioned response bias [67]. A survey of cohort studies in stroke research found that 14 of 49 (28%) articles published from 1999 to 2003 addressed potential selection bias in the recruitment of study participants and 35 (71%) mentioned the possibility that any type of bias may have affected results [5].

**Box 3. Bias**

Bias is a systematic deviation of a study's result from a true value. Typically, it is introduced during the design or implementation of a study and cannot be remedied later. Bias and confounding are not synonymous. Bias arises from flawed information or subject selection so that a wrong association is found. Confounding produces relations that are factually right, but that cannot be interpreted causally because some underlying, unaccounted for factor is associated with both exposure and outcome (see Box 5). Also, bias needs to be distinguished from random error, a deviation from a true value caused by statistical fluctuations (in either direction) in the measured data. Many possible sources of bias have been described and a variety of terms are used [68,69]. We find two simple categories helpful: information bias and selection bias.

*Information bias* occurs when systematic differences in the completeness or the accuracy of data lead to differential misclassification of individuals regarding exposures or outcomes. For instance, if diabetic women receive more regular and thorough eye examinations, the ascertainment of glaucoma will be more complete than in women without diabetes (see item 9) [65]. Patients receiving a drug that causes non-specific stomach discomfort may undergo gastroscopy more often and have more ulcers detected than patients not receiving the drug – even if the drug does not cause more ulcers. This type of information bias is also called ‘detection bias' or ‘medical surveillance bias'. One way to assess its influence is to measure the intensity of medical surveillance in the different study groups, and to adjust for it in statistical analyses. In case-control studies information bias occurs if cases recall past exposures more or less accurately than controls without that disease, or if they are more or less willing to report them (also called ‘recall bias'). ‘Interviewer bias' can occur if interviewers are aware of the study hypothesis and subconsciously or consciously gather data selectively [70]. Some form of blinding of study participants and researchers is therefore often valuable.

*Selection bias* may be introduced in case-control studies if the probability of including cases or controls is associated with exposure. For instance, a doctor recruiting participants for a study on deep-vein thrombosis might diagnose this disease in a woman who has leg complaints and takes oral contraceptives. But she might not diagnose deep-vein thrombosis in a woman with similar complaints who is not taking such medication. Such bias may be countered by using cases and controls that were referred in the same way to the diagnostic service [71]. Similarly, the use of disease registers may introduce selection bias: if a possible relationship between an exposure and a disease is known, cases may be more likely to be submitted to a register if they have been exposed to the suspected causative agent [72]. ‘Response bias' is another type of selection bias that occurs if differences in characteristics between those who respond and those who decline participation in a study affect estimates of prevalence, incidence and, in some circumstances, associations. In general, selection bias affects the internal validity of a study. This is different from problems that may arise with the selection of participants for a study in general, which affects the external rather than the internal validity of a study (also see item 21).

### 10. Study size: Explain how the study size was arrived at.

#### Example 1

“The number of cases in the area during the study period determined the sample size” [73].

#### Example 2

“A survey of postnatal depression in the region had documented a prevalence of 19.8%. Assuming depression in mothers with normal weight children to be 20% and an odds ratio of 3 for depression in mothers with a malnourished child we needed 72 case-control sets (one case to one control) with an 80% power and 5% significance” [74].

#### Explanation

A study should be large enough to obtain a point estimate with a sufficiently narrow confidence interval to meaningfully answer a research question. Large samples are needed to distinguish a small association from no association. Small studies often provide valuable information, but wide confidence intervals may indicate that they contribute less to current knowledge in comparison with studies providing estimates with narrower confidence intervals. Also, small studies that show ‘interesting' or ‘statistically significant' associations are published more frequently than small studies that do not have ‘significant' findings. While these studies may provide an early signal in the context of discovery, readers should be informed of their potential weaknesses.

The importance of sample size determination in observational studies depends on the context. If an analysis is performed on data that were already available for other purposes, the main question is whether the analysis of the data will produce results with sufficient statistical precision to contribute substantially to the literature, and sample size considerations will be informal. Formal, *a priori* calculation of sample size may be useful when planning a new study [75,76]. Such calculations are associated with more uncertainty than implied by the single number that is generally produced. For example, estimates of the rate of the event of interest or other assumptions central to calculations are commonly imprecise, if not guesswork [77]. The precision obtained in the final analysis can often not be determined beforehand because it will be reduced by inclusion of confounding variables in multivariable analyses [78], the degree of precision with which key variables can be measured [79], and the exclusion of some individuals.

Few epidemiological studies explain or report deliberations about sample size [4,5]. We encourage investigators to report pertinent formal sample size calculations if they were done. In other situations they should indicate the considerations that determined the study size (e.g., a fixed available sample, as in the first example above). If the observational study was stopped early when statistical significance was achieved, readers should be told. Do not bother readers with *post hoc* justifications for study size or retrospective power calculations [77]. From the point of view of the reader, confidence intervals indicate the statistical precision that was ultimately obtained. It should be realized that confidence intervals reflect statistical uncertainty only, and not all uncertainty that may be present in a study (see item 20).

**Box 4. Grouping**

There are several reasons why continuous data may be grouped [86]. When collecting data it may be better to use an ordinal variable than to seek an artificially precise continuous measure for an exposure based on recall over several years. Categories may also be helpful for presentation, for example to present all variables in a similar style, or to show a dose-response relationship.

Grouping may also be done to simplify the analysis, for example to avoid an assumption of linearity. However, grouping loses information and may reduce statistical power [87] especially when dichotomization is used [82,85,88]. If a continuous confounder is grouped, residual confounding may occur, whereby some of the variable's confounding effect remains unadjusted for (see Box 5) [62,89]. Increasing the number of categories can diminish power loss and residual confounding, and is especially appropriate in large studies. Small studies may use few groups because of limited numbers.

Investigators may choose cut-points for groupings based on commonly used values that are relevant for diagnosis or prognosis, for practicality, or on statistical grounds. They may choose equal numbers of individuals in each group using quantiles [90]. On the other hand, one may gain more insight into the association with the outcome by choosing more extreme outer groups and having the middle group(s) larger than the outer groups [91]. In case-control studies, deriving a distribution from the control group is preferred since it is intended to reflect the source population. Readers should be informed if cut-points are selected post hoc from several alternatives. In particular, if the cut-points were chosen to minimise a P value the true strength of an association will be exaggerated [81].

When analysing grouped variables, it is important to recognise their underlying continuous nature. For instance, a possible trend in risk across ordered groups can be investigated. A common approach is to model the rank of the groups as a continuous variable. Such linearity across group scores will approximate an actual linear relation if groups are equally spaced (e.g., 10 year age groups) but not otherwise. Il'yasova et al [92]. recommend publication of both the categorical and the continuous estimates of effect, with their standard errors, in order to facilitate meta-analysis, as well as providing intrinsically valuable information on dose-response. One analysis may inform the other and neither is assumption-free. Authors often ignore the ordering and consider the estimates (and P values) separately for each category compared to the reference category. This may be useful for description, but may fail to detect a real trend in risk across groups. If a trend is observed, a confidence interval for a slope might indicate the strength of the observation.

### 11. Quantitative variables: Explain how quantitative variables were handled in the analyses. If applicable, describe which groupings were chosen, and why.

#### Example

“Patients with a Glasgow Coma Scale less than 8 are considered to be seriously injured. A GCS of 9 or more indicates less serious brain injury. We examined the association of GCS in these two categories with the occurrence of death within 12 months from injury” [80].

#### Explanation

Investigators make choices regarding how to collect and analyse quantitative data about exposures, effect modifiers and confounders. For example, they may group a continuous exposure variable to create a new categorical variable (see Box 4). Grouping choices may have important consequences for later analyses [81,82]. We advise that authors explain why and how they grouped quantitative data, including the number of categories, the cut-points, and category mean or median values. Whenever data are reported in tabular form, the counts of cases, controls, persons at risk, person-time at risk, etc. should be given for each category. Tables should not consist solely of effect-measure estimates or results of model fitting.

Investigators might model an exposure as continuous in order to retain all the information. In making this choice, one needs to consider the nature of the relationship of the exposure to the outcome. As it may be wrong to assume a linear relation automatically, possible departures from linearity should be investigated. Authors could mention alternative models they explored during analyses (e.g., using log transformation, quadratic terms or spline functions). Several methods exist for fitting a non-linear relation between the exposure and outcome [82–84]. Also, it may be informative to present both continuous and grouped analyses for a quantitative exposure of prime interest.

In a recent survey, two thirds of epidemiological publications studied quantitative exposure variables [4]. In 42 of 50 articles (84%) exposures were grouped into several ordered categories, but often without any stated rationale for the choices made. Fifteen articles used linear associations to model continuous exposure but only two reported checking for linearity. In another survey, of the psychological literature, dichotomization was justified in only 22 of 110 articles (20%) [85].

### 12. Statistical methods: 12 (a). Describe all statistical methods, including those used to control for confounding.

#### Example

“The adjusted relative risk was calculated using the Mantel-Haenszel technique, when evaluating if confounding by age or gender was present in the groups compared. The 95% confidence interval (CI) was computed around the adjusted relative risk, using the variance according to Greenland and Robins and Robins et al.” [93].

#### Explanation

In general, there is no one correct statistical analysis but, rather, several possibilities that may address the same question, but make different assumptions. Regardless, investigators should pre-determine analyses at least for the primary study objectives in a study protocol. Often additional analyses are needed, either instead of, or as well as, those originally envisaged, and these may sometimes be motivated by the data. When a study is reported, authors should tell readers whether particular analyses were suggested by data inspection. Even though the distinction between pre-specified and exploratory analyses may sometimes be blurred, authors should clarify reasons for particular analyses.

If groups being compared are not similar with regard to some characteristics, adjustment should be made for possible confounding variables by stratification or by multivariable regression (see Box 5) [94]. Often, the study design determines which type of regression analysis is chosen. For instance, Cox proportional hazard regression is commonly used in cohort studies [95]. whereas logistic regression is often the method of choice in case-control studies [96,97]. Analysts should fully describe specific procedures for variable selection and not only present results from the final model [98,99]. If model comparisons are made to narrow down a list of potential confounders for inclusion in a final model, this process should be described. It is helpful to tell readers if one or two covariates are responsible for a great deal of the apparent confounding in a data analysis. Other statistical analyses such as imputation procedures, data transformation, and calculations of attributable risks should also be described. Non-standard or novel approaches should be referenced and the statistical software used reported. As a guiding principle, we advise statistical methods be described “with enough detail to enable a knowledgeable reader with access to the original data to verify the reported results” [100].

In an empirical study, only 93 of 169 articles (55%) reporting adjustment for confounding clearly stated how continuous and multi-category variables were entered into the statistical model [101]. Another study found that among 67 articles in which statistical analyses were adjusted for confounders, it was mostly unclear how confounders were chosen [4].

### 12 (b). Describe any methods used to examine subgroups and interactions.

#### Example

“Sex differences in susceptibility to the 3 lifestyle-related risk factors studied were explored by testing for biological interaction according to Rothman: a new composite variable with 4 categories (*a* ^−^ *b* ^−^ *, a* ^−^ *b^+^, a^+^b* ^−^ *,* and *a^+^b^+^*) was redefined for sex and a dichotomous exposure of interest where *a^−^* and *b^−^* denote absence of exposure. RR was calculated for each category after adjustment for age. An interaction effect is defined as departure from additivity of absolute effects, and excess RR caused by interaction (RERI) was calculated:

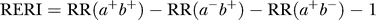
where RR(*a^+^b^+^*) denotes RR among those exposed to both factors where RR(*a* ^−^ *b* ^−^) is used as reference category (RR = 1.0). Ninety-five percent CIs were calculated as proposed by Hosmer and Lemeshow. RERI of 0 means no interaction” [103].

#### Explanation

As discussed in detail under item 17, many debate the use and value of analyses restricted to subgroups of the study population [4,104]. Subgroup analyses are nevertheless often done [4]. Readers need to know which subgroup analyses were planned in advance, and which arose while analysing the data. Also, it is important to explain what methods were used to examine whether effects or associations differed across groups (see item 17).

Interaction relates to the situation when one factor modifies the effect of another (therefore also called ‘effect modification'). The joint action of two factors can be characterized in two ways: on an additive scale, in terms of risk differences; or on a multiplicative scale, in terms of relative risk (see Box 8). Many authors and readers may have their own preference about the way interactions should be analysed. Still, they may be interested to know to what extent the joint effect of exposures differs from the separate effects. There is consensus that the additive scale, which uses absolute risks, is more appropriate for public health and clinical decision making [105]. Whatever view is taken, this should be clearly presented to the reader, as is done in the example above [103]. A lay-out presenting separate effects of both exposures as well as their joint effect, each relative to no exposure, might be most informative. It is presented in the example for interaction under item 17, and the calculations on the different scales are explained in Box 8.

**Box 5. Confounding**

Confounding literally means confusion of effects. A study might seem to show either an association or no association between an exposure and the risk of a disease. In reality, the seeming association or lack of association is due to another factor that determines the occurrence of the disease but that is also associated with the exposure. The other factor is called the confounding factor or confounder. Confounding thus gives a wrong assessment of the potential ‘causal' association of an exposure. For example, if women who approach middle age and develop elevated blood pressure are less often prescribed oral contraceptives, a simple comparison of the frequency of cardiovascular disease between those who use contraceptives and those who do not, might give the wrong impression that contraceptives protect against heart disease.

Investigators should think beforehand about potential confounding factors. This will inform the study design and allow proper data collection by identifying the confounders for which detailed information should be sought. Restriction or matching may be used. In the example above, the study might be restricted to women who do not have the confounder, elevated blood pressure. Matching on blood pressure might also be possible, though not necessarily desirable (see Box 2). In the analysis phase, investigators may use stratification or multivariable analysis to reduce the effect of confounders. Stratification consists of dividing the data in strata for the confounder (e.g., strata of blood pressure), assessing estimates of association within each stratum, and calculating the combined estimate of association as a weighted average over all strata. Multivariable analysis achieves the same result but permits one to take more variables into account simultaneously. It is more flexible but may involve additional assumptions about the mathematical form of the relationship between exposure and disease.

Taking confounders into account is crucial in observational studies, but readers should not assume that analyses adjusted for confounders establish the ‘causal part' of an association. Results may still be distorted by residual confounding (the confounding that remains after unsuccessful attempts to control for it [102]), random sampling error, selection bias and information bias (see Box 3).

### 12 (c). Explain how missing data were addressed.

#### Example

“Our missing data analysis procedures used missing at random (MAR) assumptions. We used the MICE (multivariate imputation by chained equations) method of multiple multivariate imputation in STATA. We independently analysed 10 copies of the data, each with missing values suitably imputed, in the multivariate logistic regression analyses. We averaged estimates of the variables to give a single mean estimate and adjusted standard errors according to Rubin's rules” [106].

#### Explanation

Missing data are common in observational research. Questionnaires posted to study participants are not always filled in completely, participants may not attend all follow-up visits and routine data sources and clinical databases are often incomplete. Despite its ubiquity and importance, few papers report in detail on the problem of missing data [5,107]. Investigators may use any of several approaches to address missing data. We describe some strengths and limitations of various approaches in Box 6. We advise that authors report the number of missing values for each variable of interest (exposures, outcomes, confounders) and for each step in the analysis. Authors should give reasons for missing values if possible, and indicate how many individuals were excluded because of missing data when describing the flow of participants through the study (see also item 13). For analyses that account for missing data, authors should describe the nature of the analysis (e.g., multiple imputation) and the assumptions that were made (e.g., missing at random, see Box 6).

### 12 (d). Cohort study: If applicable, describe how loss to follow-up was addressed.

#### Example

“In treatment programmes with active follow-up, those lost to follow-up and those followed-up at 1 year had similar baseline CD4 cell counts (median 115 cells per μL and 123 cells per μL), whereas patients lost to follow-up in programmes with no active follow-up procedures had considerably lower CD4 cell counts than those followed-up (median 64 cells per μL and 123 cells per μL). (…) Treatment programmes with passive follow-up were excluded from subsequent analyses” [116].

#### Explanation

Cohort studies are analysed using life table methods or other approaches that are based on the person-time of follow-up and time to developing the disease of interest. Among individuals who remain free of the disease at the end of their observation period, the amount of follow-up time is assumed to be unrelated to the probability of developing the outcome. This will be the case if follow-up ends on a fixed date or at a particular age. Loss to follow-up occurs when participants withdraw from a study before that date. This may hamper the validity of a study if loss to follow-up occurs selectively in exposed individuals, or in persons at high risk of developing the disease (‘informative censoring'). In the example above, patients lost to follow-up in treatment programmes with no active follow-up had fewer CD4 helper cells than those remaining under observation and were therefore at higher risk of dying [116].

It is important to distinguish persons who reach the end of the study from those lost to follow-up. Unfortunately, statistical software usually does not distinguish between the two situations: in both cases follow-up time is automatically truncated (‘censored') at the end of the observation period. Investigators therefore need to decide, ideally at the stage of planning the study, how they will deal with loss to follow-up. When few patients are lost, investigators may either exclude individuals with incomplete follow-up, or treat them as if they withdrew alive at either the date of loss to follow-up or the end of the study. We advise authors to report how many patients were lost to follow-up and what censoring strategies they used.

**Box 6. Missing data: problems and possible solutions**

A common approach to dealing with missing data is to restrict analyses to individuals with complete data on all variables required for a particular analysis. Although such ‘complete-case' analyses are unbiased in many circumstances, they can be biased and are always inefficient [108]. Bias arises if individuals with missing data are not typical of the whole sample. Inefficiency arises because of the reduced sample size for analysis.

Using the last observation carried forward for repeated measures can distort trends over time if persons who experience a foreshadowing of the outcome selectively drop out [109]. Inserting a missing category indicator for a confounder may increase residual confounding [107]. Imputation, in which each missing value is replaced with an assumed or estimated value, may lead to attenuation or exaggeration of the association of interest, and without the use of sophisticated methods described below may produce standard errors that are too small.

Rubin developed a typology of missing data problems, based on a model for the probability of an observation being missing [108,110]. Data are described as missing completely at random (MCAR) if the probability that a particular observation is missing does not depend on the value of any observable variable(s). Data are missing at random (MAR) if, given the observed data, the probability that observations are missing is independent of the actual values of the missing data. For example, suppose younger children are more prone to missing spirometry measurements, but that the probability of missing is unrelated to the true unobserved lung function, after accounting for age. Then the missing lung function measurement would be MAR in models including age. Data are missing not at random (MNAR) if the probability of missing still depends on the missing value even after taking the available data into account. When data are MNAR valid inferences require explicit assumptions about the mechanisms that led to missing data.

Methods to deal with data missing at random (MAR) fall into three broad classes [108,111]: likelihood-based approaches [112], weighted estimation [113] and multiple imputation [111,114]. Of these three approaches, multiple imputation is the most commonly used and flexible, particularly when multiple variables have missing values [115]. Results using any of these approaches should be compared with those from complete case analyses, and important differences discussed. The plausibility of assumptions made in missing data analyses is generally unverifiable. In particular it is impossible to prove that data are MAR, rather than MNAR. Such analyses are therefore best viewed in the spirit of sensitivity analysis (see items 12e and 17).

### 12 (d). Case-control study: If applicable, explain how matching of cases and controls was addressed.

#### Example

“We used McNemar's test, paired t test, and conditional logistic regression analysis to compare dementia patients with their matched controls for cardiovascular risk factors, the occurrence of spontaneous cerebral emboli, carotid disease, and venous to arterial circulation shunt” [117].

#### Explanation

In individually matched case-control studies a crude analysis of the odds ratio, ignoring the matching, usually leads to an estimation that is biased towards unity (see Box 2). A matched analysis is therefore often necessary. This can intuitively be understood as a stratified analysis: each case is seen as one stratum with his or her set of matched controls. The analysis rests on considering whether the case is more often exposed than the controls, despite having made them alike regarding the matching variables. Investigators can do such a stratified analysis using the Mantel-Haenszel method on a ‘matched' 2 by 2 table. In its simplest form the odds ratio becomes the ratio of pairs that are discordant for the exposure variable. If matching was done for variables like age and sex that are universal attributes, the analysis needs not retain the individual, person-to-person matching: a simple analysis in categories of age and sex is sufficient [50]. For other matching variables, such as neighbourhood, sibship, or friendship, however, each matched set should be considered its own stratum.

In individually matched studies, the most widely used method of analysis is conditional logistic regression, in which each case and their controls are considered together. The conditional method is necessary when the number of controls varies among cases, and when, in addition to the matching variables, other variables need to be adjusted for. To allow readers to judge whether the matched design was appropriately taken into account in the analysis, we recommend that authors describe in detail what statistical methods were used to analyse the data. If taking the matching into account does have little effect on the estimates, authors may choose to present an unmatched analysis.

### 12 (d). Cross-sectional study: If applicable, describe analytical methods taking account of sampling strategy.

#### Example

“The standard errors (SE) were calculated using the Taylor expansion method to estimate the sampling errors of estimators based on the complex sample design. (…) The overall design effect for diastolic blood pressure was found to be 1.9 for men and 1.8 for women and, for systolic blood pressure, it was 1.9 for men and 2.0 for women” [118].

#### Explanation

Most cross-sectional studies use a pre-specified sampling strategy to select participants from a source population. Sampling may be more complex than taking a simple random sample, however. It may include several stages and clustering of participants (e.g., in districts or villages). Proportionate stratification may ensure that subgroups with a specific characteristic are correctly represented. Disproportionate stratification may be useful to over-sample a subgroup of particular interest.

An estimate of association derived from a complex sample may be more or less precise than that derived from a simple random sample. Measures of precision such as standard error or confidence interval should be corrected using the design effect, a ratio measure that describes how much precision is gained or lost if a more complex sampling strategy is used instead of simple random sampling [119]. Most complex sampling techniques lead to a decrease of precision, resulting in a design effect greater than 1.

We advise that authors clearly state the method used to adjust for complex sampling strategies so that readers may understand how the chosen sampling method influenced the precision of the obtained estimates. For instance, with clustered sampling, the implicit trade-off between easier data collection and loss of precision is transparent if the design effect is reported. In the example, the calculated design effects of 1.9 for men indicates that the actual sample size would need to be 1.9 times greater than with simple random sampling for the resulting estimates to have equal precision.

### 12 (e). Describe any sensitivity analyses.

#### Example

“Because we had a relatively higher proportion of ‘missing' dead patients with insufficient data (38/148=25.7%) as compared to live patients (15/437=3.4%) (…), it is possible that this might have biased the results. We have, therefore, carried out a sensitivity analysis. We have assumed that the proportion of women using oral contraceptives in the study group applies to the whole (19.1% for dead, and 11.4% for live patients), and then applied two extreme scenarios: either all the exposed missing patients used second generation pills or they all used third-generation pills” [120].

#### Explanation

Sensitivity analyses are useful to investigate whether or not the main results are consistent with those obtained with alternative analysis strategies or assumptions [121]. Issues that may be examined include the criteria for inclusion in analyses, the definitions of exposures or outcomes [122], which confounding variables merit adjustment, the handling of missing data [120,123], possible selection bias or bias from inaccurate or inconsistent measurement of exposure, disease and other variables, and specific analysis choices, such as the treatment of quantitative variables (see item 11). Sophisticated methods are increasingly used to simultaneously model the influence of several biases or assumptions [124–126].

In 1959 Cornfield et al. famously showed that a relative risk of 9 for cigarette smoking and lung cancer was extremely unlikely to be due to any conceivable confounder, since the confounder would need to be at least nine times as prevalent in smokers as in non-smokers [127]. This analysis did not rule out the possibility that such a factor was present, but it did identify the prevalence such a factor would need to have. The same approach was recently used to identify plausible confounding factors that could explain the association between childhood leukaemia and living near electric power lines [128]. More generally, sensitivity analyses can be used to identify the degree of confounding, selection bias, or information bias required to distort an association. One important, perhaps under recognised, use of sensitivity analysis is when a study shows little or no association between an exposure and an outcome and it is plausible that confounding or other biases toward the null are present.

### RESULTS

The Results section should give a factual account of what was found, from the recruitment of study participants, the description of the study population to the main results and ancillary analyses. It should be free of interpretations and discursive text reflecting the authors' views and opinions.

### 13. Participants: 13 (a). Report the numbers of individuals at each stage of the study—e.g., numbers potentially eligible, examined for eligibility, confirmed eligible, included in the study, completing follow-up, and analysed.

#### Example

“Of the 105 freestanding bars and taverns sampled, 13 establishments were no longer in business and 9 were located in restaurants, leaving 83 eligible businesses. In 22 cases, the owner could not be reached by telephone despite 6 or more attempts. The owners of 36 bars declined study participation. (...) The 25 participating bars and taverns employed 124 bartenders, with 67 bartenders working at least 1 weekly daytime shift. Fifty-four of the daytime bartenders (81%) completed baseline interviews and spirometry; 53 of these subjects (98%) completed follow-up“ [129].

#### Explanation

Detailed information on the process of recruiting study participants is important for several reasons. Those included in a study often differ in relevant ways from the target population to which results are applied. This may result in estimates of prevalence or incidence that do not reflect the experience of the target population. For example, people who agreed to participate in a postal survey of sexual behaviour attended church less often, had less conservative sexual attitudes and earlier age at first sexual intercourse, and were more likely to smoke cigarettes and drink alcohol than people who refused [130]. These differences suggest that postal surveys may overestimate sexual liberalism and activity in the population. Such response bias (see Box 3) can distort exposure-disease associations if associations differ between those eligible for the study and those included in the study. As another example, the association between young maternal age and leukaemia in offspring, which has been observed in some case-control studies [131,132], was explained by differential participation of young women in case and control groups. Young women with healthy children were less likely to participate than those with unhealthy children [133]. Although low participation does not necessarily compromise the validity of a study, transparent information on participation and reasons for non-participation is essential. Also, as there are no universally agreed definitions for participation, response or follow-up rates, readers need to understand how authors calculated such proportions [134].

Ideally, investigators should give an account of the numbers of individuals considered at each stage of recruiting study participants, from the choice of a target population to the inclusion of participants' data in the analysis. Depending on the type of study, this may include the number of individuals considered to be potentially eligible, the number assessed for eligibility, the number found to be eligible, the number included in the study, the number examined, the number followed up and the number included in the analysis. Information on different sampling units may be required, if sampling of study participants is carried out in two or more stages as in the example above (multistage sampling). In case-control studies, we advise that authors describe the flow of participants separately for case and control groups [135]. Controls can sometimes be selected from several sources, including, for example, hospitalised patients and community dwellers. In this case, we recommend a separate account of the numbers of participants for each type of control group. Olson and colleagues proposed useful reporting guidelines for controls recruited through random-digit dialling and other methods [136].

A recent survey of epidemiological studies published in 10 general epidemiology, public health and medical journals found that some information regarding participation was provided in 47 of 107 case-control studies (59%), 49 of 154 cohort studies (32%), and 51 of 86 cross-sectional studies (59%) [137]. Incomplete or absent reporting of participation and non-participation in epidemiological studies was also documented in two other surveys of the literature [4,5]. Finally, there is evidence that participation in epidemiological studies may have declined in recent decades [137,138], which underscores the need for transparent reporting [139].

### 13 (b). Give reasons for non-participation at each stage.

#### Example

“The main reasons for non-participation were the participant was too ill or had died before interview (cases 30%, controls < 1%), nonresponse (cases 2%, controls 21%), refusal (cases 10%, controls 29%), and other reasons (refusal by consultant or general practitioner, non-English speaking, mental impairment) (cases 7%, controls 5%)” [140].

#### Explanation

Explaining the reasons why people no longer participated in a study or why they were excluded from statistical analyses helps readers judge whether the study population was representative of the target population and whether bias was possibly introduced. For example, in a cross-sectional health survey, non-participation due to reasons unlikely to be related to health status (for example, the letter of invitation was not delivered because of an incorrect address) will affect the precision of estimates but will probably not introduce bias. Conversely, if many individuals opt out of the survey because of illness, or perceived good health, results may underestimate or overestimate the prevalence of ill health in the population.

### 13 (c). Consider use of a flow diagram.

#### Example

**Figure:**
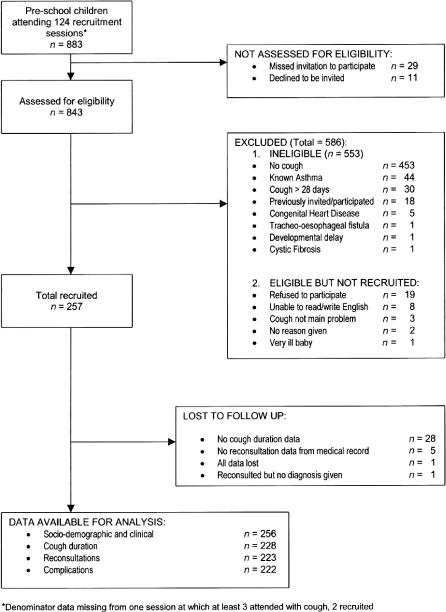
Flow diagram from Hay et al. [141].

#### Explanation

An informative and well-structured flow diagram can readily and transparently convey information that might otherwise require a lengthy description [142], as in the example above. The diagram may usefully include the main results, such as the number of events for the primary outcome. While we recommend the use of a flow diagram, particularly for complex observational studies, we do not propose a specific format for the diagram.

### 14. Descriptive data: 14 (a). Give characteristics of study participants (e.g., demographic, clinical, social) and information on exposures and potential confounders.

#### Example

[Table pmed-0040297-t002]

**Table pmed-0040297-t002:**
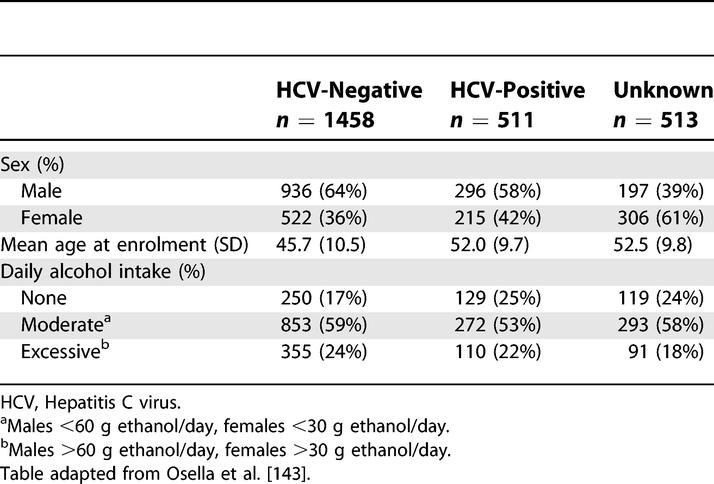
Characteristics of the Study Base at Enrolment, Castellana G (Italy), 1985–1986

#### Explanation

Readers need descriptions of study participants and their exposures to judge the generalisability of the findings. Information about potential confounders, including whether and how they were measured, influences judgments about study validity. We advise authors to summarize continuous variables for each study group by giving the mean and standard deviation, or when the data have an asymmetrical distribution, as is often the case, the median and percentile range (e.g., 25th and 75th percentiles). Variables that make up a small number of ordered categories (such as stages of disease I to IV) should not be presented as continuous variables; it is preferable to give numbers and proportions for each category (see also Box 4). In studies that compare groups, the descriptive characteristics and numbers should be given by group, as in the example above.

Inferential measures such as standard errors and confidence intervals should not be used to describe the variability of characteristics, and significance tests should be avoided in descriptive tables. Also, P values are not an appropriate criterion for selecting which confounders to adjust for in analysis; even small differences in a confounder that has a strong effect on the outcome can be important [144,145].

In cohort studies, it may be useful to document how an exposure relates to other characteristics and potential confounders. Authors could present this information in a table with columns for participants in two or more exposure categories, which permits to judge the differences in confounders between these categories.

In case-control studies potential confounders cannot be judged by comparing cases and controls. Control persons represent the source population and will usually be different from the cases in many respects. For example, in a study of oral contraceptives and myocardial infarction, a sample of young women with infarction more often had risk factors for that disease, such as high serum cholesterol, smoking and a positive family history, than the control group [146]. This does not influence the assessment of the effect of oral contraceptives, as long as the prescription of oral contraceptives was not guided by the presence of these risk factors—e.g., because the risk factors were only established after the event (see also Box 5). In case-control studies the equivalent of comparing exposed and non-exposed for the presence of potential confounders (as is done in cohorts) can be achieved by exploring the source population of the cases: if the control group is large enough and represents the source population, exposed and unexposed controls can be compared for potential confounders [121,147].

### 14 (b). Indicate the number of participants with missing data for each variable of interest.

#### Example

[Table pmed-0040297-t003]

**Table pmed-0040297-t003:**
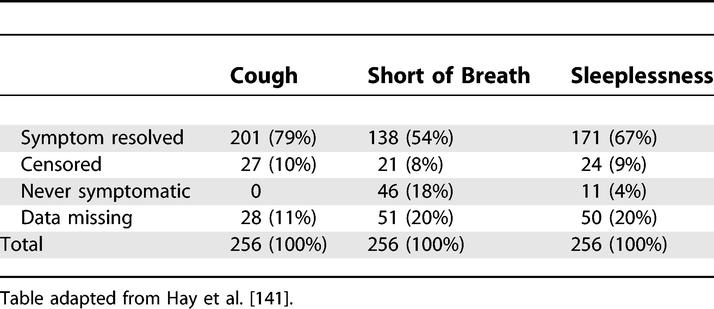
Symptom End Points Used in Survival Analysis

#### Explanation

As missing data may bias or affect generalisability of results, authors should tell readers amounts of missing data for exposures, potential confounders, and other important characteristics of patients (see also item 12c and Box 6). In a cohort study, authors should report the extent of loss to follow-up (with reasons), since incomplete follow-up may bias findings (see also items 12d and 13) [148]. We advise authors to use their tables and figures to enumerate amounts of missing data.

### 14 (c). Cohort study: Summarise follow-up time—e.g., average and total amount.

#### Example

“During the 4366 person-years of follow-up (median 5.4, maximum 8.3 years), 265 subjects were diagnosed as having dementia, including 202 with Alzheimer's disease” [149].

#### Explanation

Readers need to know the duration and extent of follow-up for the available outcome data. Authors can present a summary of the average follow-up with either the mean or median follow-up time or both. The mean allows a reader to calculate the total number of person-years by multiplying it with the number of study participants. Authors also may present minimum and maximum times or percentiles of the distribution to show readers the spread of follow-up times. They may report total person-years of follow-up or some indication of the proportion of potential data that was captured [148]. All such information may be presented separately for participants in two or more exposure categories. Almost half of 132 articles in cancer journals (mostly cohort studies) did not give any summary of length of follow-up [37].

### 15. Outcome data: Cohort study: Report numbers of outcome events or summary measures over time.

#### Example

[Table pmed-0040297-t004]

**Table pmed-0040297-t004:**
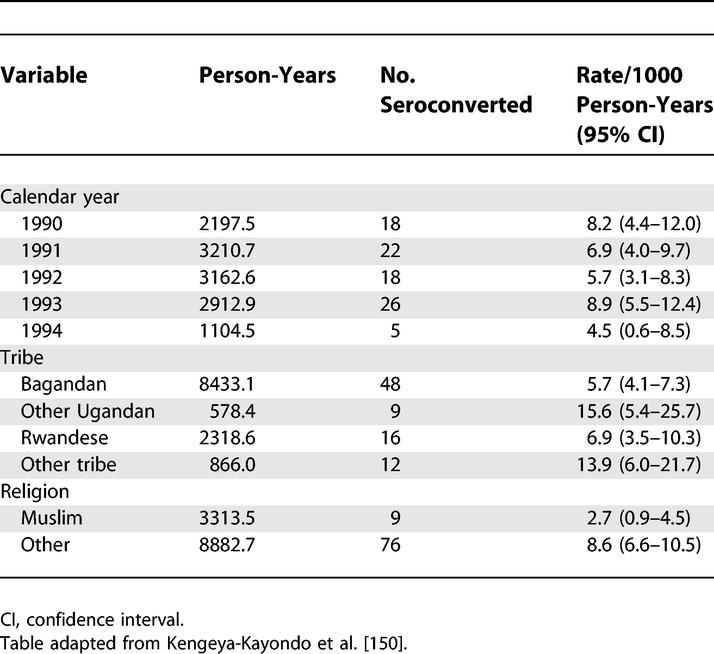
Rates of HIV-1 Seroconversion by Selected Sociodemographic Variables: 1990–1993

### Case-control study: Report numbers in each exposure category, or summary measures of exposure.

#### Example

[Table pmed-0040297-t006]

**Table pmed-0040297-t006:**
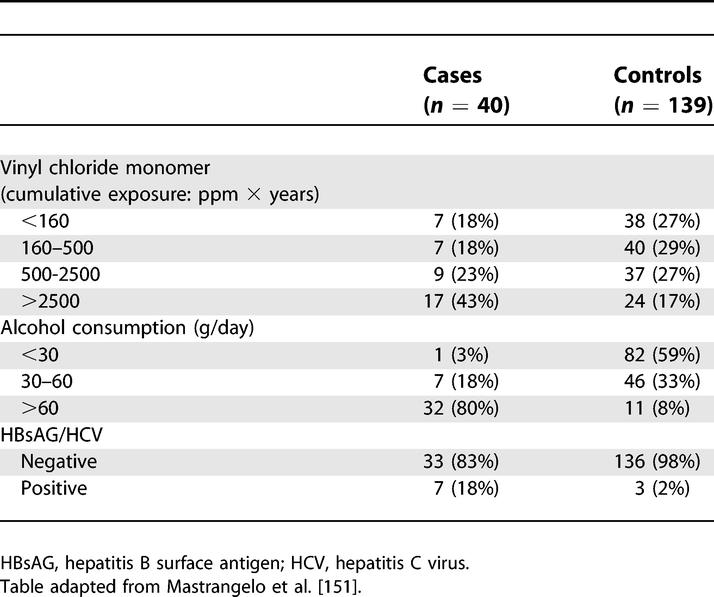
Exposure among Liver Cirrhosis Cases and Controls

### Cross-sectional study: Report numbers of outcome events or summary measures.

#### Example

[Table pmed-0040297-t007]

**Table pmed-0040297-t007:**
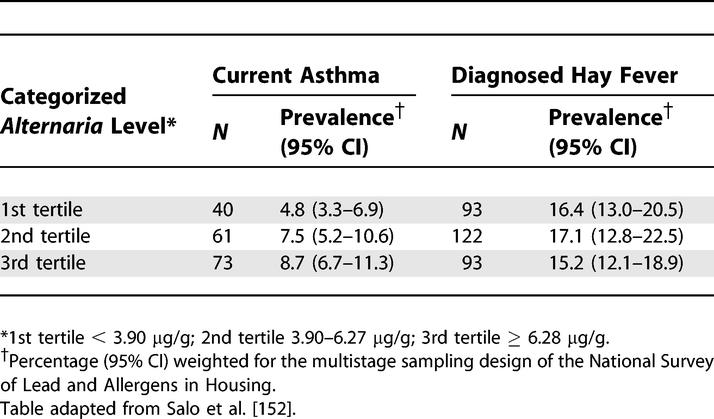
Prevalence of Current Asthma and Diagnosed Hay Fever by Average Alternaria alternata Antigen Level in the Household

#### Explanation

Before addressing the possible association between exposures (risk factors) and outcomes, authors should report relevant descriptive data. It may be possible and meaningful to present measures of association in the same table that presents the descriptive data (see item 14a). In a cohort study with events as outcomes, report the numbers of events for each outcome of interest. Consider reporting the event rate per person-year of follow-up. If the risk of an event changes over follow-up time, present the numbers and rates of events in appropriate intervals of follow-up or as a Kaplan-Meier life table or plot. It might be preferable to show plots as cumulative incidence that go up from 0% rather than down from 100%, especially if the event rate is lower than, say, 30% [153]. Consider presenting such information separately for participants in different exposure categories of interest. If a cohort study is investigating other time-related outcomes (e.g., quantitative disease markers such as blood pressure), present appropriate summary measures (e.g., means and standard deviations) over time, perhaps in a table or figure.

For cross-sectional studies, we recommend presenting the same type of information on prevalent outcome events or summary measures. For case-control studies, the focus will be on reporting exposures separately for cases and controls as frequencies or quantitative summaries [154]. For all designs, it may be helpful also to tabulate continuous outcomes or exposures in categories, even if the data are not analysed as such.

### 16. Main results: 16 (a). Give unadjusted estimates and, if applicable, confounder-adjusted estimates and their precision (e.g., 95% confidence intervals). Make clear which confounders were adjusted for and why they were included.

#### Example 1

“We initially considered the following variables as potential confounders by Mantel-Haenszel stratified analysis: (…) The variables we included in the final logistic regression models were those (…) that produced a 10% change in the odds ratio after the Mantel-Haenszel adjustment” [155].

#### Example 2

[Table pmed-0040297-t008]

**Table pmed-0040297-t008:**
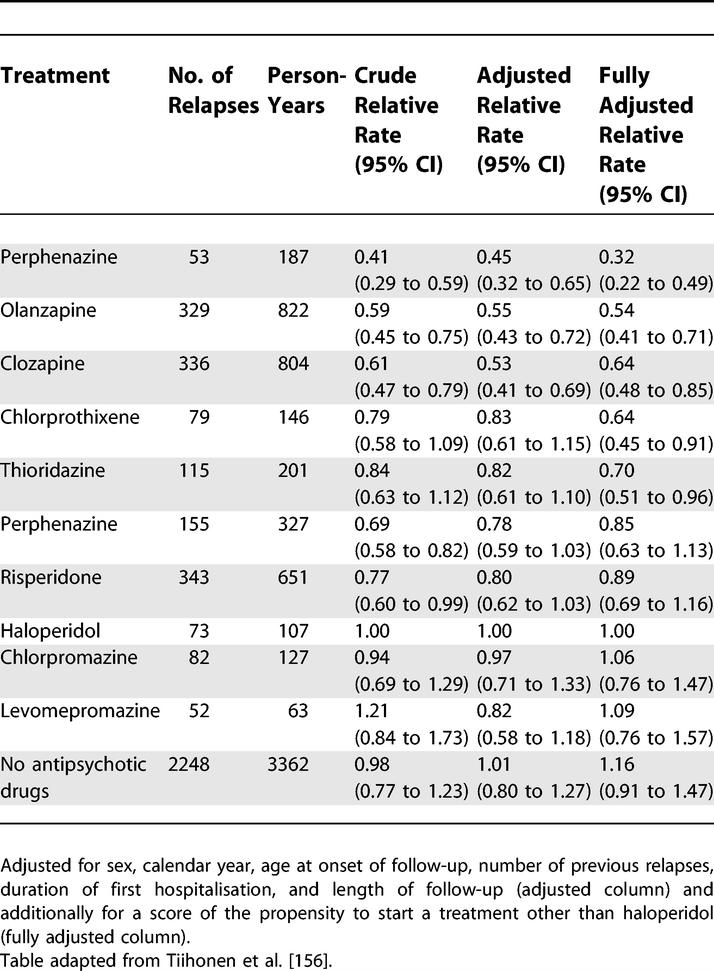
Relative Rates of Rehospitalisation by Treatment in Patients in Community Care after First Hospitalisation due to Schizophrenia and Schizoaffective Disorder

#### Explanation

In many situations, authors may present the results of unadjusted or minimally adjusted analyses and those from fully adjusted analyses. We advise giving the unadjusted analyses together with the main data, for example the number of cases and controls that were exposed or not. This allows the reader to understand the data behind the measures of association (see also item 15). For adjusted analyses, report the number of persons in the analysis, as this number may differ because of missing values in covariates (see also item 12c). Estimates should be given with confidence intervals.

Readers can compare unadjusted measures of association with those adjusted for potential confounders and judge by how much, and in what direction, they changed. Readers may think that ‘adjusted' results equal the causal part of the measure of association, but adjusted results are not necessarily free of random sampling error, selection bias, information bias, or residual confounding (see Box 5). Thus, great care should be exercised when interpreting adjusted results, as the validity of results often depends crucially on complete knowledge of important confounders, their precise measurement, and appropriate specification in the statistical model (see also item 20) [157,158].

Authors should explain all potential confounders considered, and the criteria for excluding or including variables in statistical models. Decisions about excluding or including variables should be guided by knowledge, or explicit assumptions, on causal relations. Inappropriate decisions may introduce bias, for example by including variables that are in the causal pathway between exposure and disease (unless the aim is to asses how much of the effect is carried by the intermediary variable). If the decision to include a variable in the model was based on the change in the estimate, it is important to report what change was considered sufficiently important to justify its inclusion. If a ‘backward deletion' or ‘forward inclusion' strategy was used to select confounders, explain that process and give the significance level for rejecting the null hypothesis of no confounding. Of note, we and others do not advise selecting confounders based solely on statistical significance testing [147,159,160].

Recent studies of the quality of reporting of epidemiological studies found that confidence intervals were reported in most articles [4]. However, few authors explained their choice of confounding variables [4,5].

### 16 (b). Report category boundaries when continuous variables were categorised.

#### Example

[Table pmed-0040297-t005]

**Table pmed-0040297-t005:**
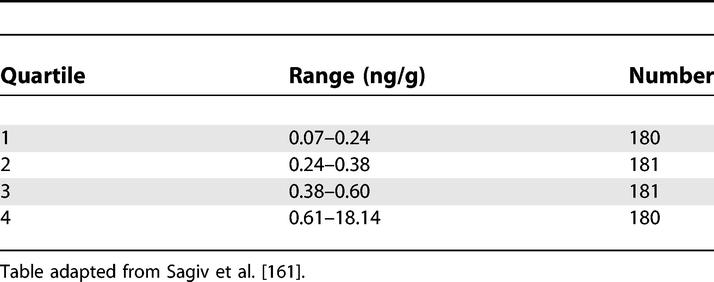
Polychlorinated Biphenyls in Cord Serum

#### Explanation

Categorizing continuous data has several important implications for analysis (see Box 4) and also affects the presentation of results. In tables, outcomes should be given for each exposure category, for example as counts of persons at risk, person-time at risk, if relevant separately for each group (e.g., cases and controls). Details of the categories used may aid comparison of studies and meta-analysis. If data were grouped using conventional cut-points, such as body mass index thresholds [162], group boundaries (i.e., range of values) can be derived easily, except for the highest and lowest categories. If quantile-derived categories are used, the category boundaries cannot be inferred from the data. As a minimum, authors should report the category boundaries; it is helpful also to report the range of the data and the mean or median values within categories.

### 16 (c). If relevant, consider translating estimates of relative risk into absolute risk for a meaningful time period.

#### Example

“10 years' use of HRT [hormone replacement therapy] is estimated to result in five (95% CI 3–7) additional breast cancers per 1000 users of oestrogen-only preparations and 19 (15–23) additional cancers per 1000 users of oestrogen-progestagen combinations” [163].

#### Explanation

The results from studies examining the association between an exposure and a disease are commonly reported in relative terms, as ratios of risks, rates or odds (see Box 8). Relative measures capture the strength of the association between an exposure and disease. If the relative risk is a long way from 1 it is less likely that the association is due to confounding [164,165]. Relative effects or associations tend to be more consistent across studies and populations than absolute measures, but what often tends to be the case may be irrelevant in a particular instance. For example, similar relative risks were obtained for the classic cardiovascular risk factors for men living in Northern Ireland, France, the USA and Germany, despite the fact that the underlying risk of coronary heart disease varies substantially between these countries [166,167]. In contrast, in a study of hypertension as a risk factor for cardiovascular disease mortality, the data were more compatible with a constant rate difference than with a constant rate ratio [168].

Widely used statistical models, including logistic [169] and proportional hazards (Cox) regression [170] are based on ratio measures. In these models, only departures from constancy of ratio effect measures are easily discerned. Nevertheless, measures which assess departures from additivity of risk differences, such as the Relative Excess Risk from Interaction (RERI, see item 12b and Box 8), can be estimated in models based on ratio measures.

In many circumstances, the absolute risk associated with an exposure is of greater interest than the relative risk. For example, if the focus is on adverse effects of a drug, one will want to know the number of additional cases per unit time of use (e.g., days, weeks, or years). The example gives the additional number of breast cancer cases per 1000 women who used hormone-replacement therapy for 10 years [163]. Measures such as the attributable risk or population attributable fraction may be useful to gauge how much disease can be prevented if the exposure is eliminated. They should preferably be presented together with a measure of statistical uncertainty (e.g., confidence intervals as in the example). Authors should be aware of the strong assumptions made in this context, including a causal relationship between a risk factor and disease (also see Box 7) [171]. Because of the semantic ambiguity and complexities involved, authors should report in detail what methods were used to calculate attributable risks, ideally giving the formulae used [172].

A recent survey of abstracts of 222 articles published in leading medical journals found that in 62% of abstracts of randomised trials including a ratio measure absolute risks were given, but only in 21% of abstracts of cohort studies [173]. A free text search of Medline 1966 to 1997 showed that 619 items mentioned attributable risks in the title or abstract, compared to 18,955 using relative risk or odds ratio, for a ratio of 1 to 31 [174].

**Box 7. Measures of association, effect and impact**

Observational studies may be solely done to describe the magnitude and distribution of a health problem in the population. They may examine the number of people who have a disease at a particular time (prevalence), or that develop a disease over a defined period (incidence). The incidence may be expressed as the proportion of people developing the disease (cumulative incidence) or as a rate per person-time of follow-up (incidence rate). Specific terms are used to describe different incidences; amongst others, mortality rate, birth rate, attack rate, or case fatality rate. Similarly, terms like point prevalence and period, annual or lifetime prevalence are used to describe different types of prevalence [30].

Other observational studies address cause-effect relationships. Their focus is the comparison of the risk, rate or prevalence of the event of interest between those exposed and those not exposed to the risk factor under investigation. These studies often estimate a ‘relative risk', which may stand for risk ratios (ratios of cumulative incidences) as well as rate ratios (ratios of incidence rates). In case-control studies only a fraction of the source population (the controls) are included. Results are expressed as the ratio of the odds of exposure among cases and controls. This odds ratio provides an estimate of the risk or rate ratio depending on the sampling of cases and controls (see also Box 1) [175,176]. The prevalence ratio or prevalence odds ratio from cross-sectional studies may be useful in some situations [177].

Expressing results both in relative and absolute terms may often be helpful. For example, in a study of male British doctors the incidence rate of death from lung cancer over 50 years of follow-up was 249 per 100,000 per year among smokers, compared to 17 per 100,000 per year among non-smokers: a rate ratio of 14.6 (249/17) [178]. For coronary heart disease (CHD), the corresponding rates were 1001 and 619 per 100,000 per year, for a rate ratio of 1.61 (1001/619). The effect of smoking on death appears much stronger for lung cancer than for CHD. The picture changes when we consider the absolute effects of smoking. The difference in incidence rates was 232 per 100,000 per year (249 − 17) for lung cancer and 382 for CHD (1001 − 619). Therefore, among doctors who smoked, smoking was more likely to cause death from CHD than from lung cancer.

How much of the disease burden in a population could be prevented by eliminating an exposure? Global estimates have been published for smoking: according to one study 91% of all lung cancers, 40% of CHD and 33% of all deaths among men in 2000 were attributed to smoking [179]. The population attributable fraction is generally defined as the proportion of cases caused by a particular exposure, but several concepts (and no unified terminology) exist, and incorrect approaches to adjust for other factors are sometimes used [172,180]. What are the implications for reporting? The relative measures emphasise the strength of an association, and are most useful in etiologic research. If a causal relationship with an exposure is documented and associations are interpreted as *effects*, estimates of relative risk may be translated into suitable measures of absolute risk in order to gauge the possible impact of public health policies (see item 16c) [181]. However, authors should be aware of the strong assumptions made in this context [171]. Care is needed in deciding which concept and method is appropriate for a particular situation.

### 17. Other analyses: Report other analyses done—e.g., analyses of subgroups and interactions, and sensitivity analyses.

#### Example 1

[Table pmed-0040297-t009]

**Table pmed-0040297-t009:**
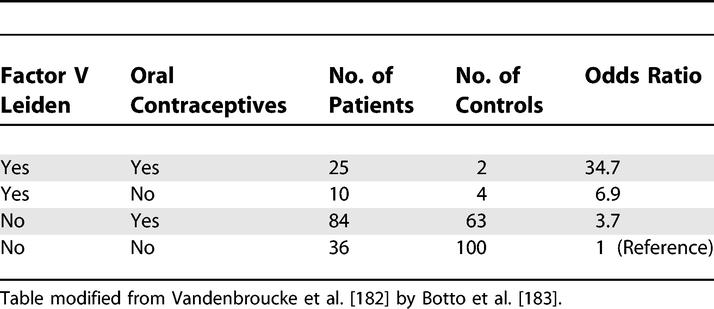
Analysis of Oral Contraceptive Use, Presence of Factor V Leiden Allele, and Risk for Venous Thromboembolism

#### Example 2

[Table pmed-0040297-t010]

**Table pmed-0040297-t010:**
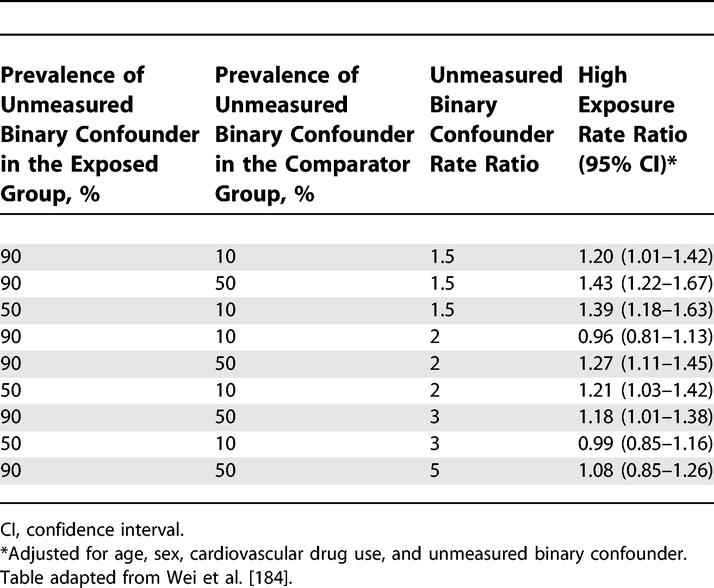
Sensitivity of the Rate Ratio for Cardiovascular Outcome to an Unmeasured Confounder

#### Explanation

In addition to the main analysis other analyses are often done in observational studies. They may address specific subgroups, the potential interaction between risk factors, the calculation of attributable risks, or use alternative definitions of study variables in sensitivity analyses.

There is debate about the dangers associated with subgroup analyses, and multiplicity of analyses in general [4,104]. In our opinion, there is too great a tendency to look for evidence of subgroup-specific associations, or effect-measure modification, when overall results appear to suggest little or no effect. On the other hand, there is value in exploring whether an overall association appears consistent across several, preferably pre-specified subgroups especially when a study is large enough to have sufficient data in each subgroup. A second area of debate is about interesting subgroups that arose during the data analysis. They might be important findings, but might also arise by chance. Some argue that it is neither possible nor necessary to inform the reader about all subgroup analyses done as future analyses of other data will tell to what extent the early exciting findings stand the test of time [9]. We advise authors to report which analyses were planned, and which were not (see also items 4, 12b and 20). This will allow readers to judge the implications of multiplicity, taking into account the study's position on the continuum from discovery to verification or refutation.

A third area of debate is how joint effects and interactions between risk factors should be evaluated: on additive or multiplicative scales, or should the scale be determined by the statistical model that fits best (see also item 12b and Box 8)? A sensible approach is to report the separate effect of each exposure as well as the joint effect—if possible in a table, as in the first example above [183], or in the study by Martinelli et al. [185]. Such a table gives the reader sufficient information to evaluate additive as well as multiplicative interaction (how these calculations are done is shown in Box 8). Confidence intervals for separate and joint effects may help the reader to judge the strength of the data. In addition, confidence intervals around measures of interaction, such as the Relative Excess Risk from Interaction (RERI) relate to tests of interaction or homogeneity tests. One recurrent problem is that authors use comparisons of P-values across subgroups, which lead to erroneous claims about an effect modifier. For instance, a statistically significant association in one category (e.g., men), but not in the other (e.g., women) does not in itself provide evidence of effect modification. Similarly, the confidence intervals for each point estimate are sometimes inappropriately used to infer that there is no interaction when intervals overlap. A more valid inference is achieved by directly evaluating whether the magnitude of an association differs across subgroups.

Sensitivity analyses are helpful to investigate the influence of choices made in the statistical analysis, or to investigate the robustness of the findings to missing data or possible biases (see also item 12b). Judgement is needed regarding the level of reporting of such analyses. If many sensitivity analyses were performed, it may be impractical to present detailed findings for them all. It may sometimes be sufficient to report that sensitivity analyses were carried out and that they were consistent with the main results presented. Detailed presentation is more appropriate if the issue investigated is of major concern, or if effect estimates vary considerably [59,186].

Pocock and colleagues found that 43 out of 73 articles reporting observational studies contained subgroup analyses. The majority claimed differences across groups but only eight articles reported a formal evaluation of interaction (see item 12b) [4].

**Box 8. Interaction (effect modification): the analysis of joint effects**

Interaction exists when the association of an exposure with the risk of disease differs in the presence of another exposure. One problem in evaluating and reporting interactions is that the effect of an exposure can be measured in two ways: as a relative risk (or rate ratio) or as a risk difference (or rate difference). The use of the relative risk leads to a multiplicative model, while the use of the risk difference corresponds to an additive model [187,188]. A distinction is sometimes made between ‘statistical interaction' which can be a departure from either a multiplicative or additive model, and ‘biologic interaction' which is measured by departure from an additive model [189]. However, neither additive nor multiplicative models point to a particular biologic mechanism.

Regardless of the model choice, the main objective is to understand how the joint effect of two exposures differs from their separate effects (in the absence of the other exposure). The Human Genomic Epidemiology Network (HuGENet) proposed a lay-out for transparent presentation of separate and joint effects that permits evaluation of different types of interaction [183]. Data from the study on oral contraceptives and factor V Leiden mutation [182] were used to explain the proposal, and this example is also used in item 17. Oral contraceptives and factor V Leiden mutation each increase the risk of venous thrombosis; their separate and joint effects can be calculated from the 2 by 4 table (see example 1 for item 17) where the odds ratio of 1 denotes the baseline of women without Factor V Leiden who do not use oral contraceptives.

A difficulty is that some study designs, such as case-control studies, and several statistical models, such as logistic or Cox regression models, estimate relative risks (or rate ratios) and intrinsically lead to multiplicative modelling. In these instances, relative risks can be translated to an additive scale. In example 1 of item 17, the separate odds ratios are 3.7 and 6.9; the joint odds ratio is 34.7. When these data are analysed under a multiplicative model, a joint odds ratio of 25.7 is expected (3.7 × 6.9). The observed joint effect of 34.7 is 1.4 times greater than expected on a multiplicative scale (34.7/25.7). This quantity (1.4) is the odds ratio of the multiplicative interaction. It would be equal to the antilog of the estimated interaction coefficient from a logistic regression model. Under an additive model the joint odds ratio is expected to be 9.6 (3.7 + 6.9 – 1). The observed joint effect departs strongly from additivity: the difference is 25.1 (34.7 – 9.6). When odds ratios are interpreted as relative risks (or rate ratios), the latter quantity (25.1) is the Relative Excess Risk from Interaction (RERI) [190]. This can be understood more easily when imagining that the reference value (equivalent to OR=1) represents a baseline incidence of venous thrombosis of, say, 1/10 000 women-years, which then increases in the presence of separate and joint exposures.

### DISCUSSION

The discussion section addresses the central issues of validity and meaning of the study [191]. Surveys have found that discussion sections are often dominated by incomplete or biased assessments of the study's results and their implications, and rhetoric supporting the authors' findings [192,193]. Structuring the discussion may help authors avoid unwarranted speculation and over-interpretation of results while guiding readers through the text [194,195]. For example, *Annals of Internal Medicine* [196] recommends that authors structure the discussion section by presenting the following: (1) a brief synopsis of the key findings; (2) consideration of possible mechanisms and explanations; (3) comparison with relevant findings from other published studies; (4) limitations of the study; and (5) a brief section that summarizes the implications of the work for practice and research. Others have made similar suggestions [191,194]. The section on research recommendations and the section on limitations of the study should be closely linked to each other. Investigators should suggest ways in which subsequent research can improve on their studies rather than blandly stating ‘more research is needed' [197,198]. We recommend that authors structure their discussion sections, perhaps also using suitable subheadings.

### 18. Key results: Summarise key results with reference to study objectives.

#### Example

“We hypothesized that ethnic minority status would be associated with higher levels of cardiovascular disease (CVD) risk factors, but that the associations would be explained substantially by socioeconomic status (SES). Our hypothesis was not confirmed. After adjustment for age and SES, highly significant differences in body mass index, blood pressure, diabetes, and physical inactivity remained between white women and both black and Mexican American women. In addition, we found large differences in CVD risk factors by SES, a finding that illustrates the high-risk status of both ethnic minority women as well as white women with low SES“ [199].

#### Explanation

It is good practice to begin the discussion with a short summary of the main findings of the study. The short summary reminds readers of the main findings and may help them assess whether the subsequent interpretation and implications offered by the authors are supported by the findings.

### 19. Limitations: Discuss limitations of the study, taking into account sources of potential bias or imprecision. Discuss both direction and magnitude of any potential bias.

#### Example

“Since the prevalence of counseling increases with increasing levels of obesity, our estimates may overestimate the true prevalence. Telephone surveys also may overestimate the true prevalence of counseling. Although persons without telephones have similar levels of overweight as persons with telephones, persons without telephones tend to be less educated, a factor associated with lower levels of counseling in our study. Also, of concern is the potential bias caused by those who refused to participate as well as those who refused to respond to questions about weight. Furthermore, because data were collected cross-sectionally, we cannot infer that counseling preceded a patient's attempt to lose weight” [200].

#### Explanation

The identification and discussion of the limitations of a study are an essential part of scientific reporting. It is important not only to identify the sources of bias and confounding that could have affected results, but also to discuss the relative importance of different biases, including the likely direction and magnitude of any potential bias (see also item 9 and Box 3).

Authors should also discuss any imprecision of the results. Imprecision may arise in connection with several aspects of a study, including the study size (item 10) and the measurement of exposures, confounders and outcomes (item 8). The inability to precisely measure true values of an exposure tends to result in bias towards unity: the less precisely a risk factor is measured, the greater the bias. This effect has been described as ‘attenuation' [201,202], or more recently as ‘regression dilution bias' [203]. However, when correlated risk factors are measured with different degrees of imprecision, the adjusted relative risk associated with them can be biased towards or away from unity [204–206].

When discussing limitations, authors may compare the study being presented with other studies in the literature in terms of validity, generalisability and precision. In this approach, each study can be viewed as contribution to the literature, not as a stand-alone basis for inference and action [207]. Surprisingly, the discussion of important limitations of a study is sometimes omitted from published reports. A survey of authors who had published original research articles in *The Lancet* found that important weaknesses of the study were reported by the investigators in the survey questionnaires, but not in the published article [192].

### 20. Interpretation: Give a cautious overall interpretation considering objectives, limitations, multiplicity of analyses, results from similar studies, and other relevant evidence.

#### Example

“Any explanation for an association between death from myocardial infarction and use of second generation oral contraceptives must be conjectural. There is no published evidence to suggest a direct biologic mechanism, and there are no other epidemiologic studies with relevant results. (…) The increase in absolute risk is very small and probably applies predominantly to smokers. Due to the lack of corroborative evidence, and because the analysis is based on relatively small numbers, more evidence on the subject is needed. We would not recommend any change in prescribing practice on the strength of these results” [120].

#### Explanation

The heart of the discussion section is the interpretation of a study's results. Over-interpretation is common and human: even when we try hard to give an objective assessment, reviewers often rightly point out that we went too far in some respects. When interpreting results, authors should consider the nature of the study on the discovery to verification continuum and potential sources of bias, including loss to follow-up and non-participation (see also items 9, 12 and 19). Due consideration should be given to confounding (item 16a), the results of relevant sensitivity analyses, and to the issue of multiplicity and subgroup analyses (item 17). Authors should also consider residual confounding due to unmeasured variables or imprecise measurement of confounders. For example, socioeconomic status (SES) is associated with many health outcomes and often differs between groups being compared. Variables used to measure SES (income, education, or occupation) are surrogates for other undefined and unmeasured exposures, and the true confounder will by definition be measured with error [208]. Authors should address the real range of uncertainty in estimates, which is larger than the statistical uncertainty reflected in confidence intervals. The latter do not take into account other uncertainties that arise from a study's design, implementation, and methods of measurement [209].

To guide thinking and conclusions about causality, some may find criteria proposed by Bradford Hill in 1965 helpful [164]. How strong is the association with the exposure? Did it precede the onset of disease? Is the association consistently observed in different studies and settings? Is there supporting evidence from experimental studies, including laboratory and animal studies? How specific is the exposure's putative effect, and is there a dose-response relationship? Is the association biologically plausible? These criteria should not, however, be applied mechanically. For example, some have argued that relative risks below 2 or 3 should be ignored [210,211]. This is a reversal of the point by Cornfield et al. about the strength of large relative risks (see item 12b) [127]. Although a causal effect is more likely with a relative risk of 9, it does not follow that one below 3 is necessarily spurious. For instance, the small increase in the risk of childhood leukaemia after intrauterine irradiation is credible because it concerns an adverse effect of a medical procedure for which no alternative explanations are obvious [212]. Moreover, the carcinogenic effects of radiation are well established. The doubling in the risk of ovarian cancer associated with eating 2 to 4 eggs per week is not immediately credible, since dietary habits are associated with a large number of lifestyle factors as well as SES [213]. In contrast, the credibility of much debated epidemiologic findings of a difference in thrombosis risk between different types of oral contraceptives was greatly enhanced by the differences in coagulation found in a randomised cross-over trial [214]. A discussion of the existing external evidence, from different types of studies, should always be included, but may be particularly important for studies reporting small increases in risk. Further, authors should put their results in context with similar studies and explain how the new study affects the existing body of evidence, ideally by referring to a systematic review.

### 21. Generalisability: Discuss the generalisability (external validity) of the study results.

#### Example

”How applicable are our estimates to other HIV-1-infected patients? This is an important question because the accuracy of prognostic models tends to be lower when applied to data other than those used to develop them. We addressed this issue by penalising model complexity, and by choosing models that generalised best to cohorts omitted from the estimation procedure. Our database included patients from many countries from Europe and North America, who were treated in different settings. The range of patients was broad: men and women, from teenagers to elderly people were included, and the major exposure categories were well represented. The severity of immunodeficiency at baseline ranged from not measureable to very severe, and viral load from undetectable to extremely high” [215].

#### Explanation

Generalisability, also called external validity or applicability, is the extent to which the results of a study can be applied to other circumstances [216]. There is no external validity *per se*; the term is meaningful only with regard to clearly specified conditions [217]. Can results be applied to an individual, groups or populations that differ from those enrolled in the study with regard to age, sex, ethnicity, severity of disease, and co-morbid conditions? Are the nature and level of exposures comparable, and the definitions of outcomes relevant to another setting or population? Are data that were collected in longitudinal studies many years ago still relevant today? Are results from health services research in one country applicable to health systems in other countries?

The question of whether the results of a study have external validity is often a matter of judgment that depends on the study setting, the characteristics of the participants, the exposures examined, and the outcomes assessed. Thus, it is crucial that authors provide readers with adequate information about the setting and locations, eligibility criteria, the exposures and how they were measured, the definition of outcomes, and the period of recruitment and follow-up. The degree of non-participation and the proportion of unexposed participants in whom the outcome develops are also relevant. Knowledge of the absolute risk and prevalence of the exposure, which will often vary across populations, are helpful when applying results to other settings and populations (see Box 7).

### OTHER INFORMATION 22. Funding: Give the source of funding and the role of the funders for the present study and, if applicable, for the original study on which the present article is based.

#### Explanation

Some journals require authors to disclose the presence or absence of financial and other conflicts of interest [100,218]. Several investigations show strong associations between the source of funding and the conclusions of research articles [219–222]. The conclusions in randomised trials recommended the experimental drug as the drug of choice much more often (odds ratio 5.3) if the trial was funded by for-profit organisations, even after adjustment for the effect size [223]. Other studies document the influence of the tobacco and telecommunication industries on the research they funded [224–227]. There are also examples of undue influence when the sponsor is governmental or a non-profit organisation.

Authors or funders may have conflicts of interest that influence any of the following: the design of the study [228]; choice of exposures [228,229], outcomes [230], statistical methods [231], and selective publication of outcomes [230] and studies [232]. Consequently, the role of the funders should be described in detail: in what part of the study they took direct responsibility (e.g., design, data collection, analysis, drafting of manuscript, decision to publish) [100]. Other sources of undue influence include employers (e.g., university administrators for academic researchers and government supervisors, especially political appointees, for government researchers), advisory committees, litigants, and special interest groups.

## Concluding Remarks

The STROBE Statement aims to provide helpful recommendations for reporting observational studies in epidemiology. Good reporting reveals the strengths and weaknesses of a study and facilitates sound interpretation and application of study results. The STROBE Statement may also aid in planning observational studies, and guide peer reviewers and editors in their evaluation of manuscripts.

We wrote this explanatory article to discuss the importance of transparent and complete reporting of observational studies, to explain the rationale behind the different items included in the checklist, and to give examples from published articles of what we consider good reporting. We hope that the material presented here will assist authors and editors in using STROBE.

We stress that STROBE and other recommendations on the reporting of research [13,233,234] should be seen as evolving documents that require continual assessment, refinement, and, if necessary, change [235,236]. For example, the CONSORT Statement for the reporting of parallel-group randomized trials was first developed in the mid 1990s [237]. Since then members of the group have met regularly to review the need to revise the recommendations; a revised version appeared in 2001 [233] and a further version is in development. Similarly, the principles presented in this article and the STROBE checklist are open to change as new evidence and critical comments accumulate. The STROBE Web site (http://www.strobe-statement.org/) provides a forum for discussion and suggestions for improvements of the checklist, this explanatory document and information about the good reporting of epidemiological studies.

Several journals ask authors to follow the STROBE Statement in their instructions to authors (see http://www.strobe-statement.org/ for current list). We invite other journals to adopt the STROBE Statement and contact us through our Web site to let us know. The journals publishing the STROBE recommendations provide open access. The STROBE Statement is therefore widely accessible to the biomedical community.

## Abbreviations

CI: confidence interval
RERI: Relative Excess Risk from Interaction
RR: relative risk
STROBE: Strengthening the Reporting of Observational Studies in Epidemiology
